# Assessing and correcting neighborhood socioeconomic spatial sampling biases in citizen science mosquito data collection

**DOI:** 10.1038/s41598-024-73416-6

**Published:** 2024-09-28

**Authors:** Álvaro Padilla-Pozo, Frederic Bartumeus, Tomás Montalvo, Isis Sanpera-Calbet, Andrea Valsecchi, John R. B. Palmer

**Affiliations:** 1 Department of Sociology, Cornell University, Uris Hall, 109 Tower Rd, Ithaca, 14853 New York United States of America; 2 Cornell Population Center, Cornell University, Martha Van Rensselaer Hall, Ithaca, 14850 New York United States of America; 3 Centre d’Estudis Avançats de Blanes (CEAB-CSIC), Spanish National Research Council, Carrer Accés Cala Sant Francesc, 14, Blanes, 17300 Girona Spain; 4 Department of Political and Social Sciences, Universitat Pompeu Fabra, Ramon Trias Fargas, 25-27, Barcelona, 08005 Barcelona Spain; 5 Institució Catalana de Recerca i Estudis Avançats (ICREA), Passeig de Lluís Companys, 23, Barcelona, 08010 Barcelona Spain; 6 Centre de Recerca Ecològica i Aplicacions Forestals (CREAF), Edifici C Facultad de ciencias y biociencias, Bellaterra, 08193 Barcelona Spain; 7 Agència de Salut Pública de Barcelona, Pl. de Lesseps, 1, Barcelona, 08023 Barcelona Spain; 8 CIBER Epidemiología y Salud Pública (CIBERESP), Instituto de Salud Carlos III, C/ Monforte de Lemos 3-5, Pabellón 11, Planta 0, Madrid, 28029 Madrid Spain; 9 Institut d’Investigació Biomédica Sant Pau, IIB St. Pau, Sant Quintí, 77-79, Barcelona, 08041 Barcelona Spain

**Keywords:** Mosquito-borne diseases, Vector control, Vector surveillance, *Aedes albopictus*, Citizen science, Social inequality, Urban ecology, Environmental social sciences

## Abstract

Climatic, ecological, and socioeconomic factors are facilitating the spread of mosquito-borne diseases, heightening the importance of vector surveillance and control. Citizen science is proving to be an effective tool to track mosquito populations, but methods are needed to detect and account for small scale sampling biases in citizen science surveillance. In this article we combine two types of traditional mosquito surveillance records with data from the Mosquito Alert citizen science system to explore the ways in which the socioeconomic characteristics of urban neighborhoods result in sampling biases in citizen scientists’ mosquito reports, while also shaping the spatial distribution of mosquito populations themselves. We use Barcelona, Spain, as an example, and focus on *Aedes albopictus*, an invasive vector species of concern worldwide. Our results suggest citizen scientists’ sampling effort is focused more in Barcelona’s lower and middle income census tracts than in its higher income ones, whereas *Ae. albopictus* populations are concentrated in the city’s upper-middle income tracts. High resolution estimates of the spatial distribution of *Ae. albopictus* risk can be improved by controlling for citizen scientists’ sampling effort, making it possible to provide better insights for efficiently targeting control efforts. Our methodology can be replicated in other cities faced with vector mosquitoes to improve public health responses to mosquito-borne diseases, which impose massive burdens on communities worldwide.

## Introduction

Climatic, ecological and socioeconomic factors are shifting and expanding the ranges, seasons, and fecundity of disease vector mosquitoes^16,55,62,73,78,79,86^, while also driving faster and earlier multiplication of pathogens^14,83^, all of which heightens the importance of vector surveillance and control efforts^18,92^. In the absence of viable vaccines, the World Health Organization (WHO) has long highlighted vector control as an essential but underutilized strategy for addressing the increasing global burden of vector-borne disease^90^, and the calls for member states to strengthen vector control and surveillance are becoming increasingly urgent^3,91,92^.

Responding to these calls requires adding new tools and new ways of thinking to traditional entomological approaches^26,58,74^. Citizen science is proving to be a particularly effective one^5,12,29,70^, so much so, that it is now being incorporated explicitly into national and local vector control strategies^60,61^. Citizen science involves participation in scientific research by members of the public who need not be professional scientists^37,41,66^. Participation ranges from “classical” scientific observation (like the century-old Christmas Bird Count), to networked citizen science that harnesses the internet and digital sensors to link participants and enable involvement in different parts of the research process^36^.

One of the main challenges of citizen science is to collect reliable data that can be used to make unbiased inferences. This is especially important for projects that rely on citizen scientists’ observations and reporting but have no participation restrictions—i.e., people can participate whenever and wherever they want. In these types of projects, it is generally necessary to distinguish between variation in the phenomenon of interest and variation in citizen scientists’ sampling effort^22^ because citizen scientists tend to sample some areas more than others^46^. For example sampling may be more intense in areas closer to participants’ homes^21^ or closer to cities or roads^9^. Sampling effort may also be mediated by a variety of other physical and socioeconomic spatial characteristics, including human population density, altitude, land cover type, degree of urbanization, and aggregate income levels of resident populations^32,81^.

From a vector surveillance perspective, this issue is crucial because human-related factors generally have the most unpredictable influence on vector dynamics in urban ecosystems^49^. A better understanding of the role of these factors in citizen science participation makes it possible to learn more about their influence on vectors themselves. The analysis of potential spatial sampling biases in mosquito vector population estimates that use data collected from citizen scientists is also crucial from a health equity perspective, as these estimates are increasingly used by local, regional, and national public health authorities as an integral part of vector surveillance and control^60^. Reducing spatial biases in these estimates allows for more efficient control interventions and targeting of surveillance, consequently reducing the risk of diseases that place massive burdens on society around the world and that widen existing health inequalities^8,56^.

In the present study, we investigate how socioeconomic census tract characteristics in Barcelona shape reporting patterns in Mosquito Alert, a networked citizen science platform used for vector mosquito surveillance^5,47,70^. We untangle the effects of census tract characteristics on participation from their effects on mosquito populations and the risk of exposure using ground truth data about the presence of mosquitoes. This allows us to reduce spatially structured socieconomic biases in estimates of mosquito distributions relied on by public health authorities.

Mosquito Alert leverages mobile phone sensors, the Internet, expert validation, and artificial intelligence to enable people without any prior training to identify and report vector mosquitoes^5,47,70^. Since its launch in 2014, the Mosquito Alert system has been operational in Barcelona^67,70^, and Barcelona’s public health agency, the *Agència de Salut Pública de Barcelona* (ASPB), has gradually incorporated it into its vector surveillance program^59,61^.

Since June 2014 Mosquito Alert has received over 197,353 reports from 67,276 citizen scientists in 183 countries. Of these reports, 54% represent adult mosquitoes, 35% mosquito bites, and 10% mosquito breeding sites. (For the latest participation statistics, updated daily, see https://labs.mosquitoalert.com/participation/.) Seventy-seven percent of adult and site reports are accompanied by at least one photograph, and all adult reports with photographs are validated within Mosquito Alert’s Digital Entolab system by entomologists who review the photographs and score each report’s probability of representing one of five vector species (or species complexes) of public health concern: *Ae. albopictus*, *Ae. aegypti*, *Ae. japonicus*, *Ae. koreicus*, or *Culex pipiens*^47^.

**Fig. 1:**
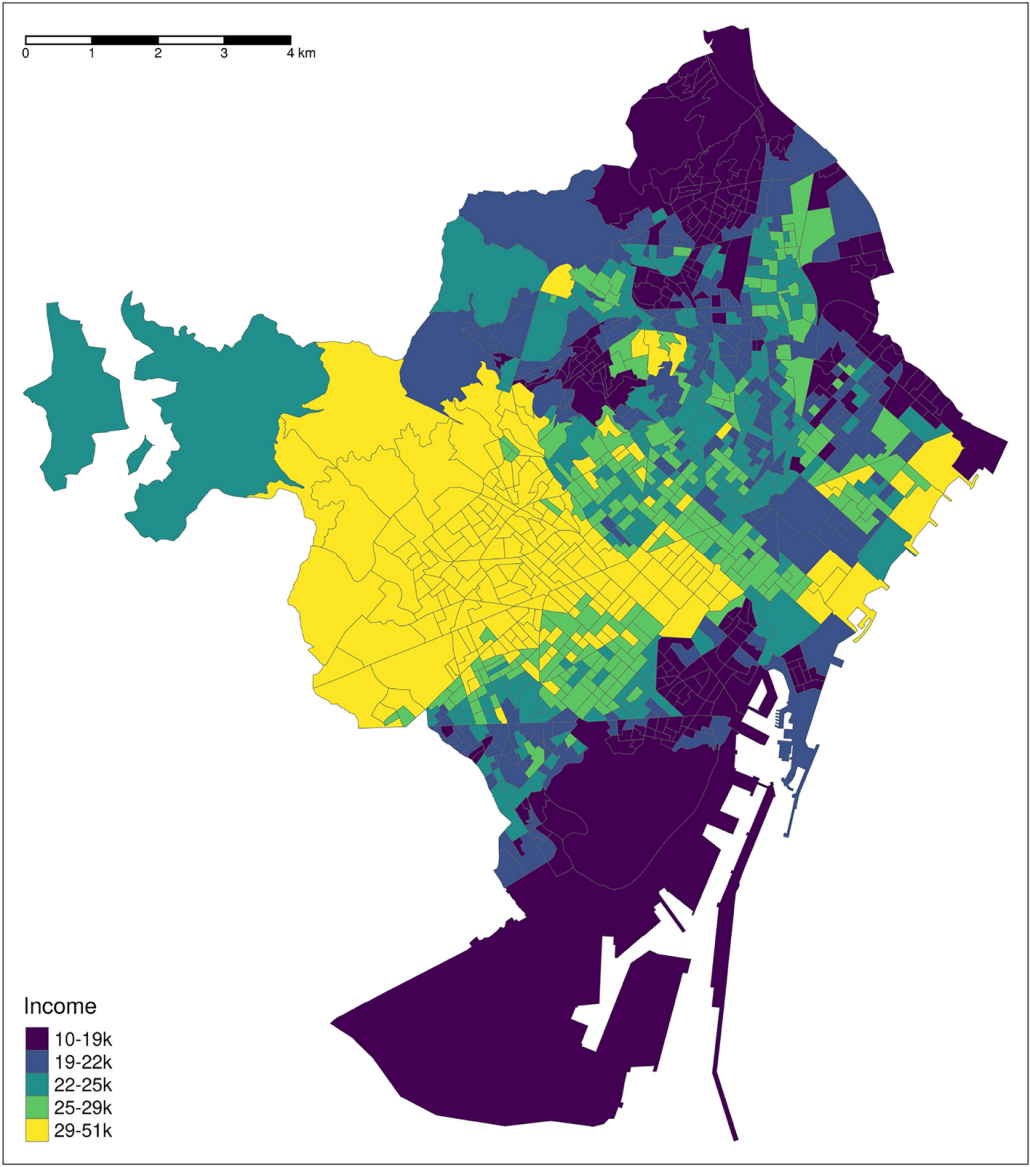
Map of Barcelona municipality showing census tracts colored by yearly net mean income per consumption unit. Cartographic image created by the authors from the the INE’s Digital Cartography Files^44^ using *R 4.4.1*^72^ with *ggplot2 3.4.4*^93^ and *ggspatial 1.1.9*^24^.

The Mosquito Alert system was designed to collect not only citizen scientists’ reports of vector mosquitoes, but also information about sampling effort that can be used to correct for bias. To protect participants’ privacy, however, its approach relies on anonymized background tracking masked to a grid of 0.025 degrees latitude and longitude (see Suppl. Info. Fig. 1)^12,47,70^. This makes it possible to estimate variation in sampling effort between cells of approximately 4 $km^2$, but not within these cells. Yet, smaller-scale variation in sampling effort may well be an important source of bias in the type of census tract-level analysis necessary for effective mosquito surveillance and control in cities.

In Barcelona, the mosquito species of primary concern is the tiger mosquito, *Ae. albopictus*, an invasive species that poses a nuisance and a public health risk through its aggressive daytime biting and its ability to transmit dengue, chikungunya, Zika and a wide range of other viruses^15,20,38,77^. Although autochthonous transmission of these viruses has not yet been detected in Barcelona, the presence of an established *Ae. albopictus* population in the city is particularly worrying given this species’ high vector competence and the city’s position as a major travel and tourism hub, with frequent cases of imported mosquito-borne infections^7,34,35,52,59^.

Barcelona contains 1068 census tracts, with important variation along many socioeconomic dimensions including income (Fig. 1). Our key variable of interest is the yearly net mean income per consumption unit of each census tract, which we use as a proxy for area-level socioeconomic status. Worldwide, evidence suggests that *Aedes* mosquitoes often cluster in poorer areas^17,76,80,87,89^ due to community features such as higher concentrations of unmanaged water containers (e.g., flower pots), overgrown vegetation, and poorly maintained or abandoned buildings^17,49,65,76,87^. Nevertheless, *Aedes* mosquito populations are also capable of clustering in census tracts with high socioeconomic status due to the availability of breeding sites created through gardening and other activities involving water inputs^49^ like swimming pools^17^. In the case of Barcelona, very little is known about the relationship between census tract socioeconomic characteristics and *Aedes* mosquito populations. Our analysis helps to address this gap for the first time, while also adding to the literature on this topic globally.

## Results

We examine citizen scientists’ reporting through Mosquito Alert in Barcelona from the time of the system’s launch in June 2014 through December 2023. This period encompasses 10 mosquito seasons, with the seasons running each year from approximately April through November. We start by examining overall participation by citizen scientists in the form of their submission of adult mosquito reports and mosquito bite reports through the system (Section "General participation model"). We then analyze citizen scientists’ Mosquito Alert reports in the immediate vicinity of catch basin drains with known mosquito activity to better isolate the socioeconomic determinants of participation levels from those of mosquito populations (Section "Active catch basin drain participation model"). We combine these results with expert-validated *Ae. albopictus* reports from Mosquito Alert to analyze the socioeconomic determinants of this species’ populations in Barcelona’s census tracts and improve high resolution estimates (Section "Mosquito Alert vector model"). Finally, we compare our findings to an independent set of estimates based on data from 51 traditional adult mosquito traps positioned around the city between 2018 and 2022 (Section "Mosquito trap vector model"). We focus our discussion on our estimated coefficients on the income variable, but we include coefficient estimates for other variables and tables of model comparisons in the Supplemental Information.

### Citizen scientist participation patterns

**Fig. 2:**
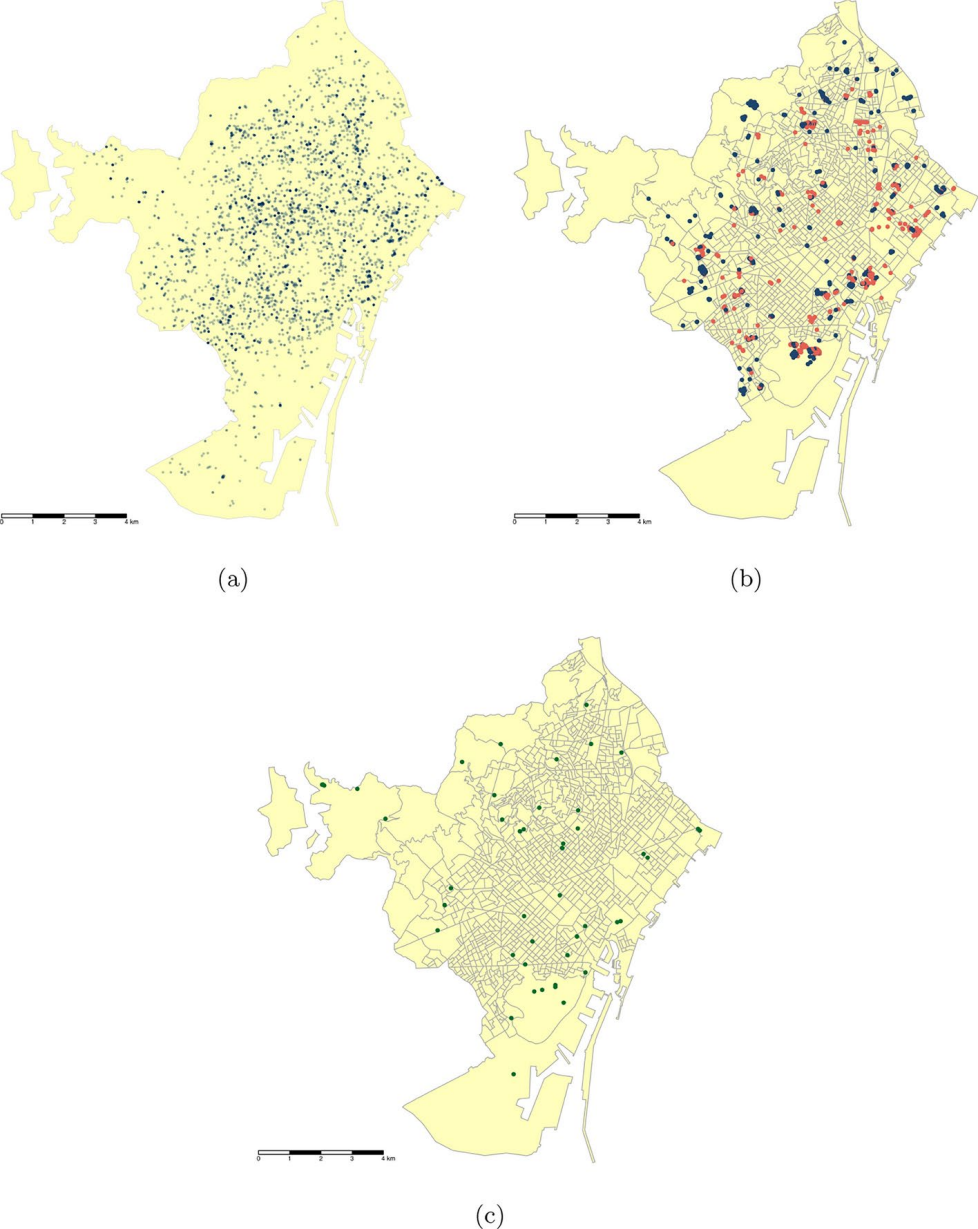
Maps of the census tracts of the municipality of Barcelona showing: (**a**) locations of adult mosquito and mosquito bite reports sent through Mosquito Alert during 2014-23, a darker shade of blue indicates a higher concentration of reports; (**b**) locations of catch basin drains in which mosquito activity was detected by ASPB during 2019-23, with colors indicating whether any (red) or no (blue) adult mosquito or mosquito bite report was sent from within 200 m of the drain during same year in which the ASPB detected the activity; (**c**) locations of adult mosquito traps from which data was collected between 2018 and 2022. Cartographic image created by the authors from the the INE’s Digital Cartography Files^44^ using *R 4.4.1*^72^ with *tmap 3.3.4*^82^.

From June 2014 through December 2023 Mosquito Alert received a total of 5,393 adult mosquito and mosquito bite reports in Barcelona (3,563 adults and 1,830 bites). These reports were heterogeneously distributed across the city and covered most of its area (79% of its census tracts). We observe a lower density of reports in the southernmost area of the city, which is an industrial and commercial area, as well as the northeastern and northern border zones of the city, which mostly consist of natural areas like a marsh and lands covered by forest (Fig. 2a).

The distribution of mean income by consumption unit (from now on referred to as “income”; we describe its calculation in the Methods section) across Barcelona’s census tracts ranges from 10,399 euros to 51,033, with a median of 23,510 (calculated in terms of the number of tracts, not people). Twenty-five percent of Barcelona’s census tracts have mean incomes below 19,970 euros; 75% have mean incomes below 27,517 euros. Spatially, income is highly autocorrelated in Barcelona (Fig. 1), with a correlation coefficient of 0.84 between the income of each census tract and that of its neighboring tracts. Overall, we find 60% of Mosquito Alert reports in census tracts above the median; we find 25% in census tracts in the highest income quartile (over 27,517 euros), and 19% in the lowest quartile (under 19,970 euros).

#### General participation model

**Fig. 3:**
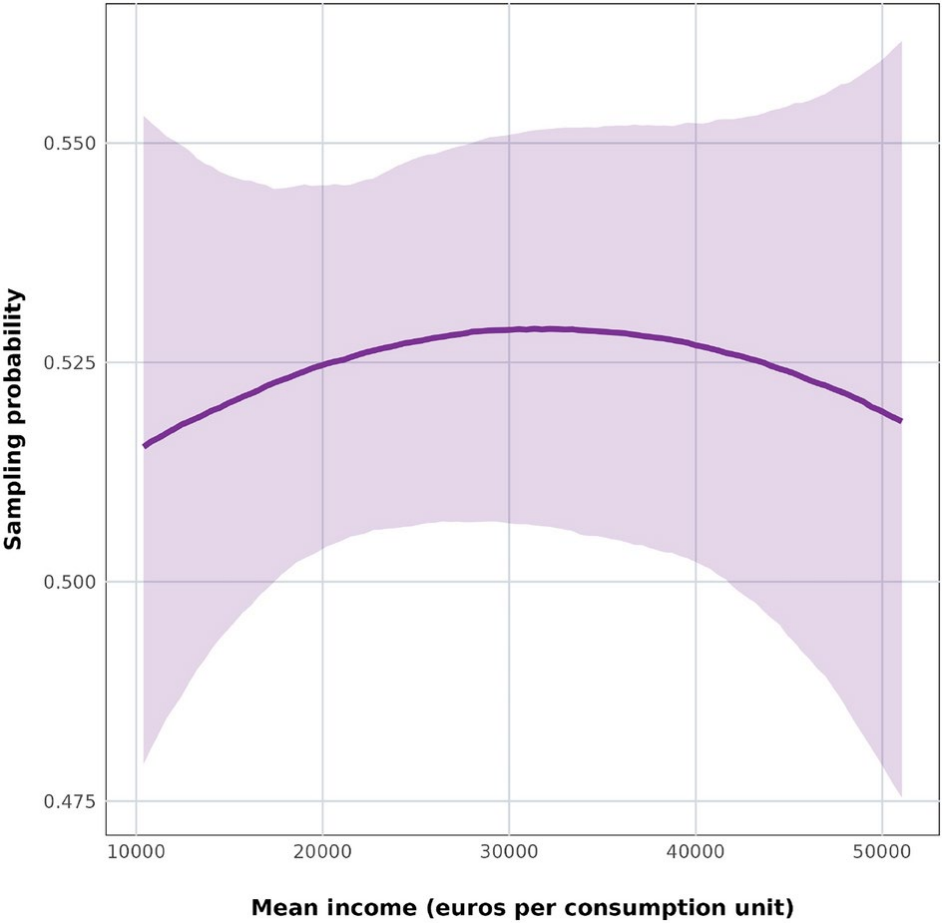
Conditional effects plot of the relationship between mean income per consumption unit (in euros) and predicted probability of citizen scientists reporting adult mosquitoes or mosquito bites in the Mosquito Alert General Participation Model, with all other variables held at their means.

**Fig. 4:**
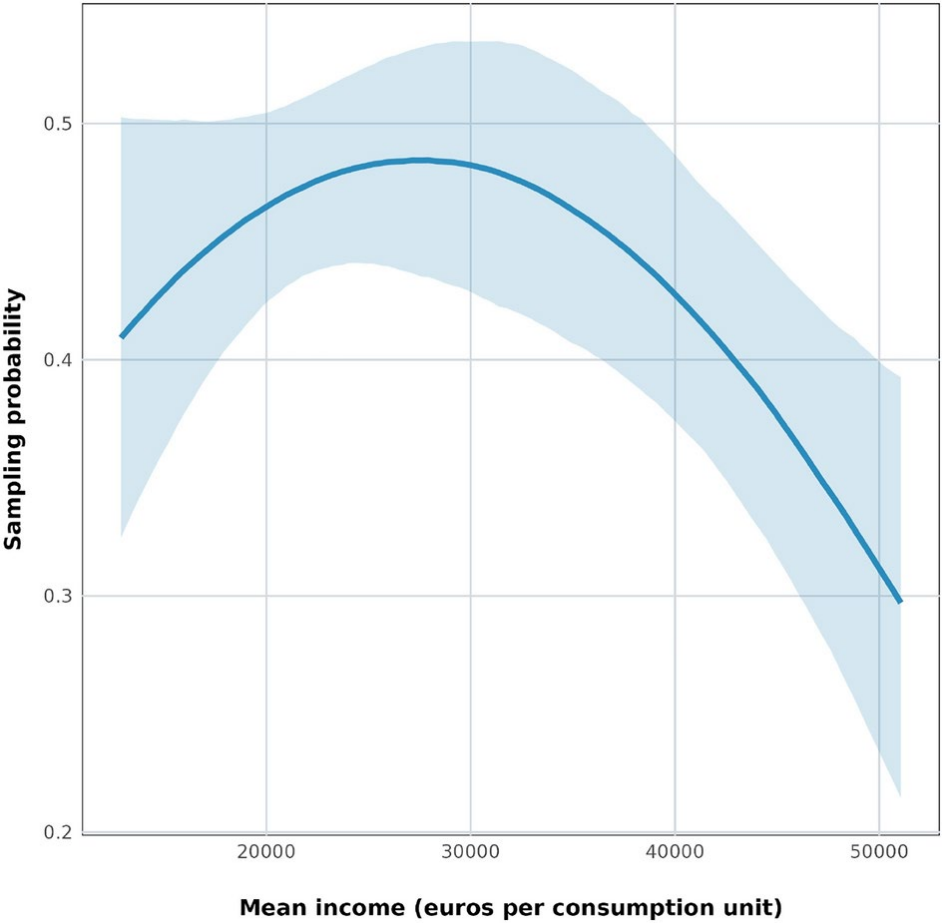
Conditional effects plot of the relationship between mean income per consumption unit (in euros) and predicted probability during a given year of citizen scientists reporting adult mosquitoes or mosquito bites within 200 m of a catch basin drain with known mosquito activity that year in the Active Catch Basin Drain Participation Model, with all other variables held at their means.

We analyze the Mosquito Alert participation data in a Bayesian logistic regression framework, modeling the log odds of a report being sent from a given point in Barcelona as a function of census tract income, population density, mean age, and proportion of households composed of only a single member (see Methods section for a description) using an intrinsic conditional auto-regressive term to account for the spatial autocorrelation of our covariates across neighboring census tracts. We transform our presence-only data (reports of mosquitoes) to a binary variable by randomly sampling pseudo-absences within the perimeter of the city of Barcelona (we describe this in detail in the Methods section). In this model (hereinafter General Participation Model) we find that, all else equal, the probability of citizen scientists’ participation has a very weak inverted-U-shaped relationship with income (Fig. 3), but there is too much uncertainty to draw firm conclusions about this relationship. We do see a clearly positive relationship between the probability of submitting a report to Mosquito Alert and population density, with the effect diminishing as density increases (Suppl. Info. Fig. 2). We also see a positive relationship with the proportion of single-member households (Suppl. Info. Fig. 3), and a negative relationship with age (Suppl. Info. Fig. 4), but in both cases there is a lot of uncertainty in the estimate. This model does not allow us to untangle whether the predicted probabilities of submitting a mosquito report are due to socioeconomic neighborhood-level processes (e.g., a higher concentration of residents with more free time), a higher (or lower) density of mosquitoes, or both. Testing this requires some independent source of information about the actual mosquito population, and for this, we turn to official mosquito surveillance and control data from the ASPB.

#### Active catch basin drain participation model

The ASPB regularly monitors mosquito activity in and around catch basin drains in public spaces of Barcelona, recording mosquito activity whenever detected^30^. We rely on the locations of 1024 drains in which the ASPB recorded mosquito activity from 2019-23. We model the presence or absence of citizen scientists’ Mosquito Alert reports within 200 m of those drains in which the ASPB detected mosquito activity during the year in which the report was sent (Fig. 2b) as a function of Mosquito Alert sampling effort (described in Methods) along with the set of independent variables used in the Mosquito Alert General Participation Model. This enables us to estimate the probability of a citizen scientist reporting mosquito activity to Mosquito Alert given that mosquito activity was actually occurring. We again use a Bayesian logistic regression, but in this case, instead of fitting a spatial autocorrelation term, we take a multilevel approach, giving each drain a random intercept to account for the fact that some of the drains are observed as active repeatedly (in multiple years). We refer to this model as our Active Catch Basin Drain Participation Model.

**Fig. 5:**
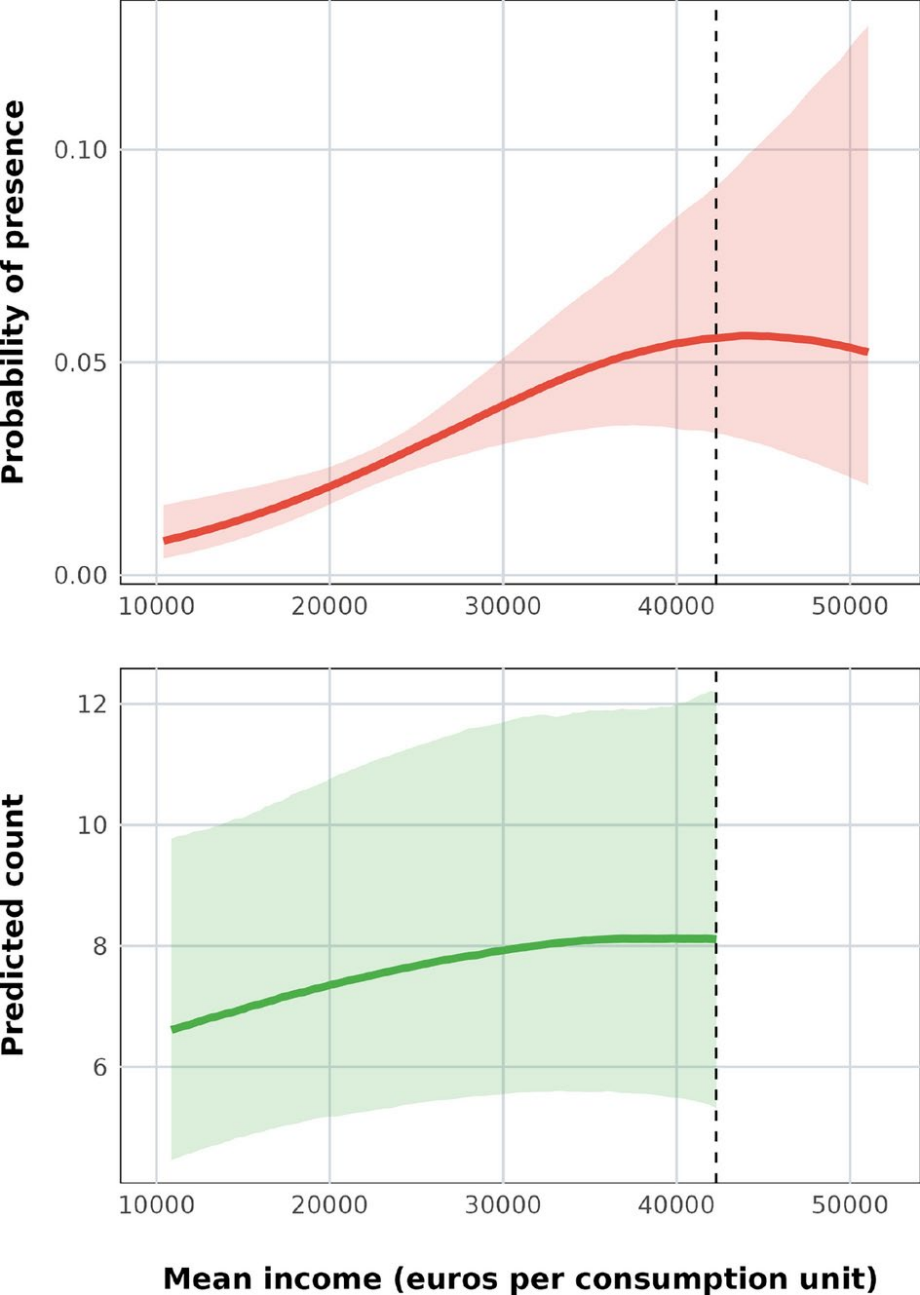
Top: Conditional effects plot of the relationship between mean income per consumption unit (in euros) and predicted *Ae. albopictus* probability in the Mosquito Alert Vector Model, with all other variables are held at their means. Bottom: Conditional effects plot of the relationship between mean income per consumption unit (in euros) and predicted *Ae. albopictus* counts in the Mosquito Trap Vector Model, with all other variables are held at their means. Dotted vertical line in both plots indicates maximum income modelled in the Mosquito Trap Vector Model.

**Fig. 6:**
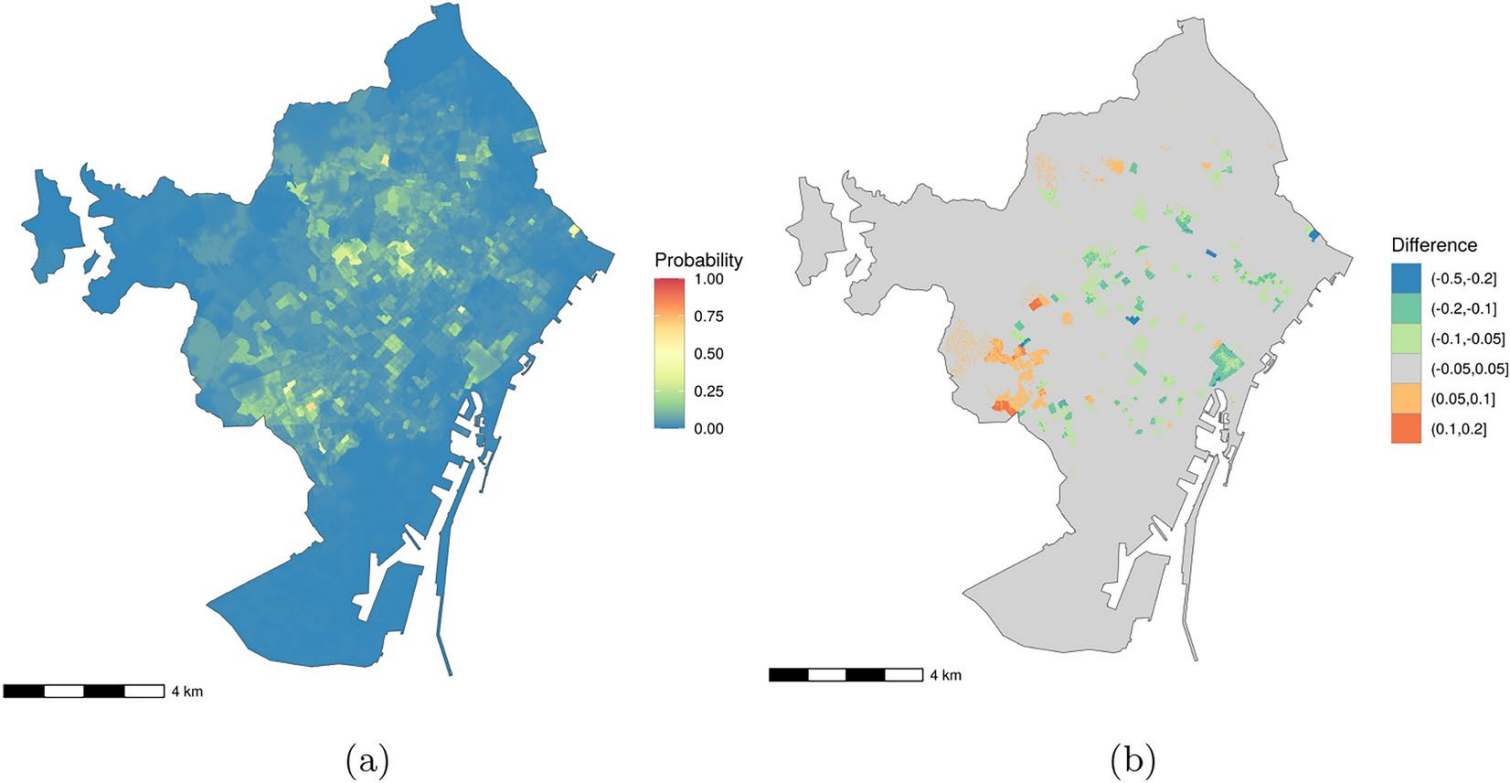
Maps of Barcelona municipality showing: (**a**) predicted probability of *Ae. albopictus* presence based on the Mosquito Alert Vector Model controlling for sampling effort, and (**b**) differences between these predictions and those made without controlling for sampling effort. The differences are calculated as predictions controlling for sampling effort minus those not controlling for sampling effort, so values above zero (oranges and reds) represent zones where probabilities would be under-predicted without controlling for sampling effort, and values below zero (greens and blues) represent zones where those probabilities would be over-predicted. Cartographic image created by the authors from the INE’s Digital Cartography Files^44^ using *R 4.4.1*^72^ with *ggplot2 3.4.4*^93^ and *ggspatial 1.1.9*^24^.

The Active Catch Basin Drain Participation Model shows a concave and mostly negative relationship between census tract income and probability of reporting (Fig. 4). We find the highest probabilities of reporting mosquitos in census tracts with incomes just below 30,000 euros (the highest income quartile among Barcelona’s census tracts), with probabilities then dropping at higher and lower incomes. Holding our other covariates at their means, the probability of a citizen scientist reporting mosquito activity near an active drain is between 40% and 50% in census tracts with incomes under 30,000 euros, and it drops to 30% in the highest-income tracts (Fig. 4). We also see the same positive, diminishing effect of population density as in the General Participation Model (Suppl. Info. Fig. 5), and here we see clear positive relationships with the proportion of households composed of a single person (Suppl. Info. Fig. 6) and with Mosquito Alert sampling effort (Suppl. Info. Fig. 7) and a clear negative relationship with age (Suppl. Info. Fig. 8).

As a robustness check, we test whether the relationships estimated in this model hold if we randomly eliminate catch basin drains from the dataset to ensure that no two modeled drains are within 200 m of one another. We find largely the same relationships in this case, albeit with more noise (Suppl. Info. Figs. 9, 10, 11, 12). The relationship between the probability of reporting a mosquito and income appears more like an inverted U in this model, showing a larger variation of sampling probability values, with much lower sampling effort in both lower and higher income tracts (Suppl. Fig. 9). Nonetheless, we attribute this to the loss of data from eliminating the drains closer than 200 m to one another.

### Vector-mosquito risk

We interpret the relationships estimated in the Active Catch Basin Drain Participation Model as reflecting sampling biases—systematic differences in the probabilities of citizen scientists reporting mosquitoes when mosquitoes are present, based on socioeconomic neighborhood characteristics. These sampling biases are important not just for what they tell us about the socioeconomic determinants of citizen science participation, but also because correcting for them should make it possible to disentangle the determinants of mosquito population sizes from the determinants of citizen scientists’ participation. In the same way that we use ASPB data on mosquito activity in the sewage system to control for variation in the actual mosquito distribution, we can use the predictions from the Active Catch Basin Drain Participation Model to control for sampling bias in a mosquito distribution model.

#### Mosquito Alert vector model

Specifically, we model the probability of *Ae. albopictus* presence, as measured through validated Mosquito Alert reports, controlling for the predicted sampling effort from our Active Catch Basin Drain Participation Model. We again rely on Bayesian logistic regressions. As in the General Participation Model, we use an intrinsic conditional auto-regressive term to account for the spatial autocorrelation of our covariates across neighboring census tracts. In this case, however, the presences in our outcome variable are locations of expert-validated *Ae. albopictus* reports (as opposed to all adult and bite reports, regardless of validation status, as modeled in the General Participation Model). In this case we generate pseudo-absences by sampling random points across Barcelona with sampling weights proportional to the sampling probabilities predicted from the Active Catch Basin Drain Participation Model. In addition to using the predicted sampling probabilities as sampling weights, we also use the log of these probabilities as an offset in the model to control for any extra socioeconomic bias for which the sampling weights do not account. Finally, we control for proximity to privately owned spaces with green land cover, a variable that the ASPB regularly uses in its mosquito surveillance and control planning based on habitat suitability of green land cover and the difficulty of carrying out mosquito control in privately-owned spaces due to lack of access.

This model estimates the relationship between census tract mean income and the probability of *Ae. albopictus* presence to be concave, but mostly increasing (Fig. 5). Holding the other variables at their means, the probability is lowest, below 1%, in the lowest-income census tracts. It increases to a maximum of just over 5% in census tracts with mean incomes between 40,000 and 50,000 euros and then decreases beyond that point, dropping to 5% in the highest-income tracts. The model also estimates a clear positive relationship between the probability of *Ae. albopictus* presence and proximity to privately owned spaces with green land cover (Suppl. Info. Fig. 13).

Figure 6 shows the predicted probabilities from this model on a regular lattice of 256,898 points across Barcelona, spaced at 20 m intervals, and it compares these predictions to those from the same model without controlling for sampling effort. The map in Figure 6a shows the predictions from the model with sampling effort, i.e., the predicted probabilities of reporting a mosquito from our Active Catch Basin Drain Participation Model, while the one in Figure 6b shows the differences between these predictions and those from the model without sampling effort, subtracting the no-sampling-effort model predictions from the sample-effort-model predictions. In this second map, the orange and red areas are those in which the model without sampling effort under-predicts the probability of *Ae. albopictus* presence, while the green and blue ones are those in which it over-predicts the probability. The model without sampling effort mostly over-predicts the probability of *Ae. albopictus* presence. It does so by more than 10 percentage points in 9 out of Barcelona’s 10 districts (Suppl. Info. Tab. 1). Under prediction by more than 10 percentage points is seen in only 3 of the 10 districts (Suppl. Info. Tab. 1).

#### Mosquito trap vector model

As a final step, we partially validate these results by testing the relationship between census tract income and a total of 1368 female *Ae. albopictus* counts that we recorded in 51 BG Sentinel-2 adult mosquito traps placed around Barcelona between 2018 and 2022 (Fig. 2c). These counts and the sampling process on which they are based have no connection to citizen scientists and so provide an independent source of information about the mosquito distribution in Barcelona. Controlling for the effects of weather (since traps were checked at different times throughout the mosquito season) and land cover, we find a similar relationship with income as in the Mosquito Alert Vector Model: The predicted counts of female *albopictus* in traps go up as we move from the lowest income census tracts to the tracts with mean incomes of 40,000 euros (Fig. 5). The predictions appear to peak at slightly lower incomes than in the Mosquito Alert Vector Model, but they are also much more noisy (Fig. 5). We hypothesize that this is due to the fact that there were very few traps in high income tracts: only 89 out of 1368 (6.5%) trap counts are from traps in tracts with mean income over 35,000 euros, and none are over 42,283. In contrast, the Mosquito Alert Vector Model is estimated using reports (both presences and pseudo-absences) from tracts with mean incomes up to 51,000.

## Discussion

These results show how spatially structured socioeconomic characteristics shape sampling bias in the Mosquito Alert citizen science reporting in Barcelona. In addition, they show how a better understanding of that bias makes it possible to learn more about the socioeconomic determinants of the small-scale spatial distribution of disease vector mosquitoes and to improve our predictive models of mosquito populations at high resolution (20m).

We find a predominantly negative relationship between census tract mean income and the probability of submitting a mosquito report to Mosquito Alert (Fig. 4). Once we control for this sampling bias, the modeled citizen science data shows a positive relationship between census tract mean income and *Ae. albopictus* probabilities up to incomes above 40,000 euros, which is largely the same as the relationship we find when we model mosquito trap data. Beyond these tract mean incomes the citizen science data suggests that probabilities begin to decrease, but there is little mosquito trap data with which to make a comparison at this income range values. Note also that 40,000 euros is at the top of Barcelona’s census tract mean income distribution, only 5% of Barcelona’s census tracts have higher mean incomes.

Whereas citizen science often faces criticism for insufficiently including communities with lower resources^42,71^, our results suggest that citizen science mosquito sampling effort in Barcelona is actually higher in these areas than in others. We hypothesize that higher-income census tracts in Barcelona have lower probabilities of submitting reports to Mosquito Alert because their residents are less exposed to mosquitoes, irrespective of mosquito prevalence, because they have higher access to air conditioning, private transportation, mosquito window nets, mosquito repellents, indoor working environments, and other amenities that reduce human-mosquito contact.

From a health and spatial inequality perspective, hazards are theorized to accumulate in poorer areas as a reflection of inequality processes at the individual level as well as structural decisions to organize space in ways that perpetuate inequality^23,33,40^. Empirical evidence supports this idea worldwide, e.g.^13,94^, even in spaces that make substantive efforts to make themselves more equitable^85^. This is also the case in Barcelona when it comes to hazards such as air pollution^53^.

Our models suggest a more complicated story when it comes to vector mosquitoes, with higher prevalence in wealthier areas. Although this is not the most common distribution pattern observed worldwide, our results emphasize the heterogeneous and location-specific nature of the association between socioeconomic status and *Aedes* mosquito presence that has been recently highlighted by other scholars^88^. In the case of Barcelona, the positive association between tract-level income and *Ae. albopictus* presence makes sense since higher income housing in Barcelona is characterized by having higher access to outdoor private spaces like terraces and gardens, where mosquitoes can proliferate easily. This does not mean that mosquito-borne disease infections should be expected to follow the same pattern as mosquito vector prevalence. Were an outbreak to occur in Barcelona, many factors could cause transmission to amplify in different parts of the city. For example, human mobility flows can facilitate the rapid spread of infected people in the viremic phase, moving the virus between mosquito subpopulations across neighborhoods^1^. Moreover, disease transmission depends not just on the density of mosquito populations but also on mosquito behavior, the density of human populations and on individuals’ exposure to mosquito bites.

Notably, the Agència de Salut Pública de Barcelona (ASPB) is making an effort to monitor mosquito presence in the city and avoid the production of mosquito-related health inequalities. In its latest report^61^, ASPB found that 59% of potential mosquito risk factors (e.g., public catch basins or fountains) were in the low-income areas of the city. Consequently, the ASPB decided to increase monitoring of mosquito activity and related risk factors in these areas, which now represent 67% of its monitoring activity^61^. This may well explain our finding, from both the Mosquito Alert Vector model and the Mosquito Trap Vector model, that *Ae. albopictus* probabilities are lower in the low-income areas.

The prevalence of *Ae. albopictus* in higher-income census tracts also suggests a mechanism by which this species’ population may be supported in Barcelona: metapopulation stability. A set of interconnected *Ae. albopictus* subpopulations utilizing different types of breeding sites, some in public spaces and others in private spaces, is more likely to survive and rebound after concerted control programs^1^. For example, when control programs focus on public spaces, they may reduce the subpopulations there, but those subpopulations can be replenished through in-migration from subpopulations in private spaces. Even with their limited dispersal capacity, *Ae. albopictus* individuals can cross the boundaries between high and low-income areas by flying between adjacent sites, through human-aided dispersal in vehicles^28,54^, or through slower multi-generational processes.

In addition to these findings about citizen science participation and the spatial distribution of mosquito populations, our approach here offers a reliable and replicable method for other citizen science projects that collect anonymized data to assess their socioeconomic spatial sampling biases as long as they have access to some source of ground truth data about the presence of the phenomenon they study. Studying the socioeconomic distribution of the underlying phenomena observed by citizen scientists in any field is crucial, as it can overlap with detrimental outcomes and trigger a process of widening social inequality.

## Methods

The core of the data for this study consists of anonymous geolocated reports sent by citizen scientists through the Mosquito Alert citizen science system^5,47,70^. We combine these reports with data from mosquito traps that we collected during 2018-22, data from the ASPB’s surveillance of catch basin drains during 2019-23, sociodemographic data from the Spanish National Statistical Institute (https://www.ine.es) and the Barcelona City Council’s Open Data Portal (https://opendata-ajuntament.barcelona.cat), and weather data from the Meteorological Service of Catalonia.

We estimate all models using Hamiltonian Monte Carlo Markov chain sampling with the no U-turn sampler implemented by *brms 2.19.0*^10,11^, *cmdstanr 0.7.1*, and *cmdstan 2.32.2*^31^ in *R 4.4.1*^72^. We use weakly informative priors for our population effects and estimate complete posterior distributions for each parameter through 4 parallel chains and 2000 iterations.

### Mosquito Alert data

We rely on descriptive content of the Mosquito Alert reports and the report dates and locations, along with optional anonymous background tracking masked to a grid of 0.025 degrees latitude and longitude (approx. 4 $km^{2}$, see Supplemental Fig. 1). Anyone can participate in Mosquito Alert as a citizen scientist if they are 18 or older. Citizen scientists participate by using a mobile phone app to report adult mosquitoes, breeding sites, and mosquito bites. Participants can optionally include photographs with adult mosquito reports, and these photographs are reviewed by a large international team of entomologists, who classify them by target species using Mosquito Alert’s digital Entolab^47^. Mosquito Alert operates around the world and collaborates with local and national public health agencies to ensure that the vector intelligence it produces is useful and actionable^12,29,47,60,74^. The system avoids collecting socio-demographic or economic information about participants for privacy reasons. However, a recent analysis of a voluntary sample of citizen scientists who had registered to use Mosquito Alert in Spain from August 2020 to September 2021 showed that participants are more likely to be women, middle-aged (30-49), employed, high earners, and live in urban spaces^19^. The tendency of higher earners to participate in Mosquito Alert in Spain as a whole contrasts with the lower propensity to report mosquitoes in the highest income areas of Barcelona. Notably, the first finding is based on a voluntary sample of the Mosquito Alert citizen scientist pool all over Spain who had begun participating in 2020-21, whilst the findings in this paper are based on mosquito bite and mosquito reports collected specifically in Barcelona and over a much wider time period (2014-23). Together, these results suggest that participation “profiles” may vary within countries and over time.

The present study uses 3563 reports of adult mosquitoes and 1830 reports of mosquito bites collected from Barcelona Municipality from June 2014 through December 2023 through Mosquito Alert^69^. The study also relies on a subset of 992 of the adult mosquito reports that contained photographs assessed by a team of expert entomologists as showing *Ae. albopictus*. That subset was derived from the full dataset of expert-validated adult reports downloaded on 20 February 2024^75^. Barcelona is the city with the highest concentration of reports in the Mosquito Alert system, in part because it is where the system was first launched and has received press attention for the longest time.

Mosquito Alert sampling effort data is taken from the open data published by Mosquito Alert daily and downloaded on 20 February 2024^64^. Specifically, we rely on the SE_expected variable in the sampling_effort_daily_cellres_025.csv.gz dataset. The dataset provides values on a grid of 0.025 degrees longitude and latitude (see Suppl. Info. Fig. 1). Each cell in the grid is referred to as a sampling cell, and it is used to protect the anonymity of the sampling effort data collected from participants^70^. The SE_expected variable represents the estimated expected number of participants sending at least one report from each sampling cell during each day from 2018 to present. The estimates are calculated by first estimating the discrete hazard function^25^ for participants’ reporting over time since registering with Mosquito Alert. In other words, we calculate the empirical probability of participants sending a report through Mosquito Alert on each day since they register, treating reporting as a repeated event^2^. We then apply this empirical probability to each participant in the background tracking data to estimate their probability of sending a report on the date in question. For the SE_expected variable, we aggregate these probabilities by summing them by sampling cell (see Suppl. Info. Fig. 1) and by day to get the expected number of participants sending at least one report from each cell each day. In other words, SE_expected for sampling cell *c* on day *d* is:

1 $$\begin{aligned} {\text {SE}}\_{\text {expected}}_{cd} = \sum _{i=1}^{N_{cd}}P(x_{icd}) \end{aligned}$$

where $P(x_{icd})$ is the probability of participant *i* sending at least one report from cell *c* on day *d* and $N_{cd}$ is the total number of participants observed in cell *c* on day *d*.

We incorporate this variable into the dataset used here by further aggregating each cell by year, summing the daily expected values to get the expected number of participants sending at least one report from each cell each year.

### Mosquito trap data

We rely on data that we collected from BG Sentinel-2 mosquito traps with BG lures (BioGents Corporation, Regensbourg, Germany). These traps attract adult, host-seeking female mosquitoes using air currents, visual queues, and (optionally) chemical queues, and capture them with a small suction fan^6,48,51^. We deployed 51 traps, between 12 and 24 per year, over four years and four mosquito seasons in cooperation with the ASPB. Trap locations are shown in Fig. 2c. Each trap was checked and mosquitoes were classified and counted approximately every week over the course of each mosquito season. There are a total of 1368 trap observations, 453 in 2018, 165 in 2020, 426 in 2021, and 324 in 2022.

### Catch basin drain data

The drain data were collected by the ASPB from 2019 to 2023 as part of its continuous surveillance and control of mosquitoes in the city^30^. This involves inspecting possible breeding sites in public spaces and applying anti-larval treatments as needed. The triggers for these inspections include direct communications about mosquito nuisance from city residents, Mosquito Alert reporting, and communications of arboviruses cases from relevant authorities, i.e. humans infected with mosquito-born viruses. Some drain inspections are also part of regular surveillance that is carried out in different risk areas of the city (around 80 areas each year). These risk areas are inspected from April to November with an inspection frequency per drain of approximately 1 visit every 3 weeks. In these inspections, all possible sources of breeding sites found in public spaces (mainly streets and roads) are checked (mainly catch basins but also ornamental fountains or other elements that can accumulate water), and data is collected on whether or not mosquito activity is detected.

### Socio-demographic data

Our principal socioeconomic indicator in all of our models is the yearly net mean income per consumption unit of each census tract in 2021, measured in euros, obtained from the most recent atlas of income distribution provided by the Spanish National Statistics Institute (INE)^43^. This variable is calculated as the sum of net incomes generated in a household divided by the units of consumption living in it. Adults are assigned a weight of one unit of consumption, individuals 14-18 years old are given a weight of 0.5, and individuals younger than 14 are assigned a weight of 0.3^43^. Adjusting household incomes by consumption unit in this way is intended to provide a value that can be more meaningfully compared across families by correcting for differences in consumption needs^95^. This variable is correlated with standard measures of inequality such as the Gini index, with a Pearson correlation coefficient of 0.54.

We obtain the percentage of census tract households composed of a single member and mean age of the census tract residential population from INE’s atlas of income distribution^43^. We estimate the population density of each census tract by dividing each tract’s population by its area, in square kilometers, which we obtain from INE’s cartography of census tracts^44^.

### Landcover data

We use data provided by the ASPB on green areas located in private spaces. This data is generated from Normalized Difference Vegetation Index (NDVI) values taken from 2019 orthophoto data available from the Cartographic and Geographic Institute of Catalonia^45^, cropped to private spaces in Barcelona. The ASPB uses this dataset as part of its surveillance planning based on its experience that these private green spaces serve as important mosquito breeding sites, particularly when breeding sites in public areas are being effectively suppressed. The data consists of a set of polygons indicating private green spaces in Barcelona, and we use this to generate a proximity variable calculated based on the distances between each observation and the closest green area. We transform these distances into a Green Proximity Index (GPI) using the following standard distance decay function:

2 $$\begin{aligned} \text {GPI} = e^{-a*d^b} \end{aligned}$$

where *d* is distance in meters and we set constants *a* to 0.01 and *b* to 1. This function takes the value of 1 when the distance is 0, and decreases with greater distances, quickly approaching zero at distances beyond 200 m, which corresponds roughly to the maximum flight-based dispersal distance of *Ae. albopictus*^57^.

### Weather data

Our Mosquito Trap Vector Model relies on historical temperature data obtained from the Meteorological Service of Catalonia. We calculate the 30-day average temperature of each trap observation using data from the five weather stations across Barcelona. The average takes the 30 days up to and including each day on which the trap was checked. We label this variable TMP.

### General participation model

Our first model (General Participation Model) estimates the probability of finding any type of mosquito report (adult or bite) in a given point location, conditional on the mean income, population density, mean age, and percentage of households composed of a single person for the census tract in which the point lies. We include population density to account for potential differences in participation across census tracts due to the amount of people living in them–assuming that higher-density areas have higher levels of participation. We include the percentage of single person households to account for the possibility that individuals living alone may have more time and motivation to participate in citizen science. We include mean age of the census tract population to account for the possibility that younger people may be more likely to participate using smartphone technology and may be more aware of Mosquito Alert.

We analyze a total of 5,393 reports (adult mosquitoes pooled with mosquito bites) covering 10 mosquito seasons from 2014 to 2023. Initially, our data is presence-only: We know locations where mosquitoes have been observed, but we have no direct data about locations in which a citizen scientist looked for mosquitoes but did not observe them. This limits our ability to make comparisons across spaces and it is a common challenge in species distribution models^27^. We deal with this by randomly sampling pseudo-absences from a uniform distribution within the perimeter of Barcelona municipality. We then generate a binary variable (1 for the presence of a report and 0 for a pseudo-absence), which we use as the outcome in our model. To minimize the impact of the influence of the distribution of our pseudo-absences in our models we sample a large number of pseudo-absences equal to our number of presences and we weight both of them equally. This approach has been shown to provide the best predictive distributions for regression-like models^4^. Our final sample consists of 10,786 observations, with the same number of presences and pseudo-absences.

The General Participation Model is a logistic regression that includes an intrinsically conditional autoregressive (ICAR) logistic term^63^ based on neighboring census tracts. ICAR models assume full spatial autocorrelation, which is empirically consistent with the overall levels of Moran’s I observed in our covariates. We treat each observation *i* as the realization of a Bernoulli random variable with mean $\pi _{i}$, and we model $\pi _{i}$ as:

3 $$\begin{aligned} \log \left( \frac{\pi _{i}}{1 - \pi _{i})}\right) = \alpha + \beta _1\text {INC}_i + \beta _2\text {INC}^2_i + \beta _3\log (\text {POP}_i) + \beta _4\text {PSH}_i + \beta _5\text {AGE}_i + Zu \end{aligned}$$

where $Z$ represents a symmetric (if $i \sim j\ \text {then } j \sim i$) and positive spatial effects matrix specified using first-order queen contiguity, with random effects modeled as a multivariate Normal distribution: $u \sim ~ N (0, \sigma ^2_u {\scriptstyle \sum })$. In other words, each census tract is neighbors with all the census tracts with which it shares a border. The covariates in this model are census tract mean income per consumption unit (INC), population density (POP), proportion of households composed of a single member (PSH), and mean population age (AGE). The $\beta$ terms represent the estimated coefficients on each variable. Table 2 in the Supplemental Information shows the degree of collinearity across our covariates, which is low and does not threaten our inferences.

Table 3 in the Supplemental Information section provides coefficient estimates for this model (GPM5) along with estimates for alternative specifications. GPM1-GPM3 take the same approach to spatial autocorrelation as GPM5 but include different combinations of covariates: GPM1 includes only mean income, GPM2 includes mean income and population density, GPM3 includes mean income, population density and mean age. GPM4 has the same covariates as GPM5 but it does not control for spatial autocorrelation. All five models have Bayes R-squared values of 0.35 and all have essentially the same expected log pointwise predictive density (ELPD) based on leave-one-out cross validation^84^ (the small differences in ELPD are overwhelmed by the ELPD standard errors). We focus on GMP5 in our analysis in order to highlight the estimated coefficients on all variables of interest.

Figure 16 in the Supplemental Information shows the prior predictive check of this model, plotting the observed number of presences and pseudo absences against the mean and central 90% of the posterior predictive distribution.

### Active catch basin drain participation model

Our second model (Active Catch Basin Drain Participation Model) estimates the probability of finding an adult mosquito or mosquito bite report in a given year within a 200-meter buffer of each drain with confirmed mosquito activity that year, conditional on the same covariates as in our first model along with Mosquito Alert sampling effort. The unit of analysis in this model is a drain-year, with a total of 1437 drain-years in which ASPB detected mosquito activity (Figure 2b). Citizen scientists sent adult mosquito reports or mosquito bite reports through Mosquito Alert from within 200 m of 623 of these active drains during the years in which they were reported as active. As with the first model, we treat each observation *i* as the realization of a Bernoulli random variable with mean $\pi _{i}$. In order to account for the autocorrelation between observations that took place at the same drains in different years (n = 74), we give random intercepts to each drain. Thus, we model $\pi _{i}$ as:

4 $$\begin{aligned} \begin{aligned} \log \left( \frac{\pi _{ij}}{1 - \pi _{ij})}\right) =~&\alpha + \beta _1\text {INC}_{ij} + \beta _2\text {INC}^2_{ij} + \beta _3\log (\text {POP})_{ij} + \beta _4\text {PSH}_{ij} \\&+ \beta _5\text {AGE}_{ij} + \beta _6\text {SE}_{ij} + \delta _j \end{aligned} \end{aligned}$$

where $\delta _j$ represents the random intercept for each active catch basin drain and SE represents the Mosquito Alert annual sampling effort for the sampling cell in which the point drain lies. As in the first model, the covariates in this model are mean income per consumption unit (INC), population density (POP), proportion of single-member households (PSH), and mean age (AGE), with the $\beta$ terms representing the estimated coefficient on each variable. Supplemental Table 4 shows the degree of collinearity across our covariates, which is low and does not threaten our inferences.

After fitting this model, we predict the probability of a citizen scientist reporting an adult mosquito or mosquito bite within 200 m of each of 256,898 points on a lattice across Barcelona spaced at 20m intervals, assuming each point represented an active catch basin drain (i.e. that mosquitoes were actually present). Each point is assigned INC, POP, PSH, AGE, and SE values based on its location. We take these predictions as a higher resolution measure of sampling effort than the one represented by the gridded Mosquito Alert sampling effort data (which uses sampling cells of approx. 4 $km^{2}$), and we use them in the next model.

Table 5 in the Supplemental Information section provides coefficient estimates for this model (ACBDPM6) along with estimates for alternative specifications. ACBDPM1-ACBDPM4 include drain-level random intercepts just like ACBDPM6, but they include different combinations of covariates: ACBDPM1 includes only mean income, ACBDPM2 includes mean income and Mosquito Alert sampling effort, ACBDPM3 includes mean income, Mosquito Alert sampling effort, and population density, ACBDPM4 includes mean income, Mosquito Alert sampling effort, population density, and proportion of households composed of a single member, and ACBDPM5 includes mean income, Mosquito Alert sampling effort, population density, proportion of households composed of a single member, and mean age. ACBDPM5 has the same covariates as ACBDPM6 but it does not include any random intercepts. ACBDPM6 has the highest Bayes R-squared value (0.14) and the second highest ELPD. The model with the highest ELPD is ACBDPM5, but the difference between the two is negligible (under 2) and overwhelmed by the ELPD standard error. That ACBDPM5 and ACBDPM6 are indistinguishable in terms of predictive accuracy indicates that the random effects of ACBDPM6 do not actually improve the model—which makes sense given that there are only 74 drains that were active in multiple years and are, thus, observed more than once in the data. We focus on ACBDPM6 in our analysis simply to show that the results are not being driven by these repeated observations.

Figure 17 in the Supplemental Information shows the prior predictive check of this model, plotting the observed number of presences and pseudo absences against the mean and central 90% of the posterior predictive distribution.

### Mosquito Alert vector model

Our third model (Mosquito Alert Vector Model) takes the same presence-pseudo-absence approach as the first model, but in this model we sample pseudo-absences from the 256,898 prediction points described above, with sampling weights proportional to the predicted sampling effort at each point. This third model estimates the probability of finding an expert-validated adult *Ae. albopictus* report, meaning a report with a photograph scored by the Entolab team as likely showing *Ae. albopictus*, conditional on the predicted sampling effort (SE) from the Active Catch Basin Drain Model, mean income per consumption unit (INC), and Green Proximity Index (GPI). SE is included as an offset (i.e. its coefficient is forced to 1) to control for the sampling bias that is not adequately accounted for by the sampling weights used for the pseudoabsences.

INC is our primary variable of interest and is expected to impact mosquito populations primarily through breeding site availability due to things like water inputs and the built environment. GPI is included to capture proximity to habitat often suitable for mosquito breeding sites.

The Mosquito Alert Vector Model is a logistic regression model with an intrinsically conditional autoregressive (ICAR) logistic term. It follows the same structure as our Active Catch Basin Drain Participation model, but without random intercepts because there are no repeated observations here. We treat each observation *i* as the realization of a Bernoulli random variable with mean $\pi _{i}$, and we model $\pi _{i}$ as:

5 $$\begin{aligned} \log \left( \frac{\pi _{i}}{1 - \pi _{i})}\right) = \alpha + \beta _1\text {INC}_i + \beta _2\text {INC}_i^2 + \beta _3\text {GPI}_i + \log (\text {SE}_i) + Zu \end{aligned}$$

where INC represents mean income per consumption unit, SE represents predicted sampling effort, GPI represents the Green Proximity Index, $Z$ represents our spatial effects matrix with random effects *u* as in the General Participation Model, and the $\beta$ terms represent the estimated coefficients on each variable. We do not include population density in this model because doing so worsened the fit of our models. Notably, the inclusion of this variable does not alter the size and direction of our predictors.

After fitting this model, we use it to make predictions on lattice of points spaced at 20 m intervals across Barcelona, based on the values of INC, log(SE), and GPI measured at each of these points.

Table 6 in the Supplemental Information section provides coefficient estimates for this model (MAVM4) along with estimates for alternative specifications. MAVM1-MAVM2 control for spatial autocorrelation, like MAVM4, using an intrinsic conditional autoregressive term, but they include different combinations of covariates: MAVM1 includes only mean income, and MAVM2 includes only the green proximity index. MAVM3 has the same covariates as MAVM4 but it does not include any spatial autocorrelation term. MAVM1, MAVM2, and MAVM4 (the models that control for spatial autocorrelation) have the same and highest Bayes R-squared value (0.21). They also have essentially the same ELPD, clearly higher than that of the non-spatial model (MAVM3). We focus on MAVM4 in our analysis in order to highlight the estimated effects of both income and green proximity, even though the addition of income to the model does not clearly improve its predictive accuracy.

Figure 18 in the Supplemental Information shows the prior predictive check of this model, plotting the observed number of presences and pseudo absences against the mean and central 90% of the posterior predictive distribution.

### Mosquito trap vector model

Finally, our fourth model (Mosquito Trap Vector Model) estimates the counts of adult *Ae. albopictus* females captured in the mosquito traps described above. Each trap-count is treated as the unit of analysis, with a total of 1368 trap-counts analyzed. We used a multilevel zero-inflated Poisson regression^39,50^ in which the outcome variable is the count of adult female *Ae. albopictus* individuals found in each trap observation. The model uses trap-level random intercepts to account for the effects of the fixed trapping sites in which traps were placed. The zero-inflated Poisson model treats the observed count *i* from trap *j* as the manifestation of random variable $Y_{ij}$, which has the probability of taking value *k* given by:

6 $$\begin{aligned} \text {Pr}(Y_ij = k) = {\left\{ \begin{array}{ll} \pi _{ij} + (1-\pi _ij)e^{-\lambda _{ij}}, & \text {if k}\ =0 \\ (1-\pi _{ij})e^{-\lambda _{ij}}\lambda _{ij}^k/k!, & \text {if k}\ >0 \end{array}\right. } \end{aligned}$$

It then estimates $\pi _{ij}$ in a logistic regression specified as:

7 $$\begin{aligned} \log \left( \frac{\pi _{ij}}{1 - \pi _{ij}}\right) = \alpha + \beta _1\text {TMP}_{ij} + \beta _2\text {TMP}_{ij}^2 \end{aligned}$$

And it estimates $\lambda _{ij}$ as a Poisson regression specified as:

8 $$\begin{aligned} \log (\lambda _{ij})= \alpha + \beta _1\text {TMP}_{ij} + \beta _2\text {TMP}_{ij}^2 + \beta _3\text {INC}_{ij} + \beta _4\text {INC}_{ij}^2 + \delta _j \end{aligned}$$

where INC and TMP are the same as above, and where $\delta _j$ represents the random intercept for trap *j*.

Table 7 in the Supplemental Information section provides coefficient estimates for this model (MTVM4) along with estimates for alternative specifications. MTVM1-MTVM2 include trap-level random intercepts like MTVM4, but MTVM1 does not include a zero-inflation term, while MTVM2 does not include the income covariates. MTVM3 includes the same zero-inflation terms and covariates as MTVM4 but does not include random intercepts. MAVM1, MAVM2, and MAVM4 have the same and highest Bayes R-squared value (0.37), wheraes MTVM3 has a much lower Bayes R-squared value (0.05). MAVM4 has the highest ELPD, clearly higher than that of MTVM1 and MTVM3 but not distinguishable from MTVM2 once we take the ELPD standard error into account. We focus on MAVM4 in our analysis in order to highlight the estimated effects of income even though the addition of income to the model does not clearly improve its predictive accuracy.

Figure 19 in the Supplemental Information shows the prior predictive check of this model, plotting the kernel density estimates of the observed trap counts against the posterior predictive distribution.

## Supplementary Information

Supplementary Information.

## Data Availability

All of the Mosquito Alert data used in this article is available in open access repositories hosted on Zenodo. The Mosquito Alert reporting data (all adult mosquito and mosquito bite reports as well as all expert-validated *Ae. albopictus* reports) is available at https://zenodo.org/doi/10.5281/zenodo.10684356^69^. The Mosquito Alert sampling effort data is available at https://doi.org/10.5281/zenodo.10674718. We have also made the BG trap and catch basin drain data available at https://zenodo.org/doi/10.5281/zenodo.10684356. We have not, however, released the exact locations of the BG traps because some of them were hosted in private spaces. The weather data relied on here was taken from the Meteorological Service of Catalonia’s API (https://apidocs.meteocat.gencat.cat). Some of this data can also be accessed through the Barcelona Open Data Portal (https://opendata-ajuntament.barcelona.cat/data/dataset/mesures-estacions-meteorologiques). Income and population data are available from the INE’s *Atlas de distribución de renta de los hogares*^43^. Census section geographic data is available from the INE’s Digital Cartography Files^44^. The open code described below includes functions to merge all of these datasets. The ASPB data on private green spaces is derived from orthophoto-based NVDI data^45^ available through the Barcelona Open Data Portal (https://opendata-ajuntament.barcelona.cat/data/en/dataset/cobertura-vegetal-ndvi/resource/0003c7ee-9b66-43f6-b9cc-ff9314c208e4). That dataset has been cropped by the city using a shapefile of private spaces that we do not have permission to make public. The maps shown in Figures 1, 2, and 6, and in Figure 1 of the Supplemental Information were produced by the authors with polygon data from the INE’s Digital Cartography Files^44^ using *ggplot2 3.4.4*^93^ and *ggspatial 1.1.9*^24^ (Figs. 1 and 6, and Suppl. Info. Fig. 1), and using *tmap 3.3.4*^82^ (Fig. 2).

## Code availability

All code necessary for replicating this analysis is available on GitHub at https://github.com/h-mip/citsci-spatial-bias and archived on Zenodo at https://zenodo.org/doi/10.5281/zenodo.10796324^68^.
