# Supplementary material for: Assessing and correcting neighborhood socioeconomic spatial sampling biases in citizen science mosquito data collection
Source: Sci Rep. 2024 Sep 28;14:22462. doi: 10.1038/s41598-024-73416-6 (PMC11439082; doi:10.1038/s41598-024-73416-6)
Supplement: Supplementary file 1 — Supplementary Information. [file 41598_2024_73416_MOESM1_ESM.pdf]

SUPPLEMENTAL INFORMATION

Assessing and Correcting Neighborhood Socioeconomic Spatial Sampling Biases in Citizen Science Mosquito Data Collection

Álvaro Padilla-Pozo[1,2,3,4*], Frederic Bartumeus[3,5,6], Tomás Montalvo[7,8,9], Isis Sanpera-Calbet[4], Andrea Valsecchi[7], John R.B. Palmer[4*]

[1]Department of Sociology, Cornell University, Uris Hall, 109 Tower Rd, Ithaca, 14853, New York, United States of America.
[2]Cornell Population Center, Cornell University, Martha Van Rensselaer Hall, Ithaca, 14850, New York, United States of America.
[3]Centre d'Estudis Avançats de Blanes (CEAB-CSIC), Spanish National Research Council, Carrer Accés Cala Sant Francesc, 14, Blanes, 17300, Girona, Spain.
[4]Department of Political and Social Sciences, Universitat Pompeu Fabra, Ramon Trias Fargas, 25-27, Barcelona, 08005, Barcelona, Spain.
[5]Institució Catalana de Recerca i Estudis Avançats (ICREA), Passeig de Lluís Companys, 23, Barcelona, 08010, Barcelona, Spain.
[6]Centre de Recerca Ecològica i Aplicacions Forestals (CREAF), Edifici C Facultad de ciencias y biociencias, Bellaterra, 08193, Barcelona, Spain.
[7]Agència de Salut Pública de Barcelona, Pl. de Lesseps, 1, Barcelona, 08023, Barcelona, Spain.
[8]CIBER Epidemiología y Salud Pública (CIBERESP), Instituto de Salud Carlos III, C/ Monforte de Lemos 3-5, Pabellón 11, Planta 0, Madrid, 28029, Madrid, Spain.
[9]Institut d'Investigació Biomédica Sant Pau, IIB St. Pau, Sant Quintí, 77-79, Barcelona, 08041, Barcelona, Spain.

*Corresponding author(s). E-mail(s): ap963@cornell.edu; john.palmer@upf.edu;

Contributing authors: fbartu@ceab.csic.es; tmontal@aspb.cat;
isis.sanpera@upf.edu; avalsecc@aspb.cat;

1 Supplemental Information Figures

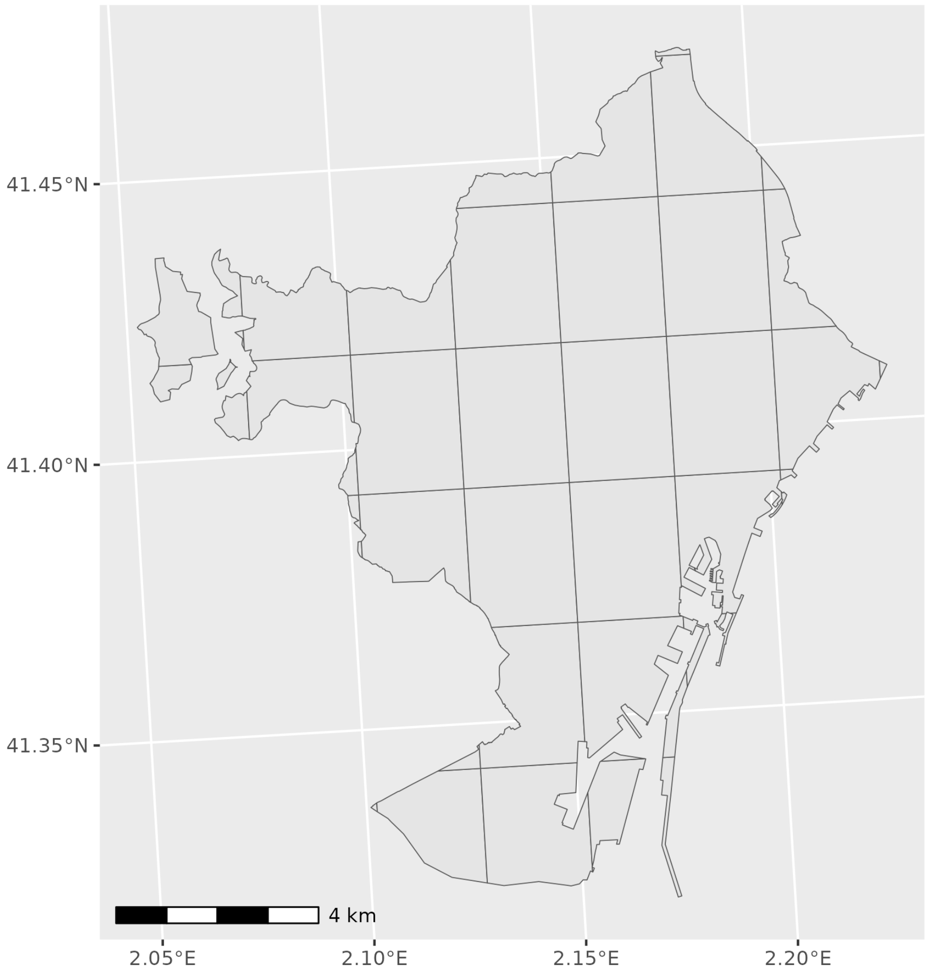

Fig. 1: Map of Barcelona municipality showing the grid of Mosquito Alert sampling cells, spaced at 0.025 degrees latitude and longitude, in which the system collects optional anonymized background tracks. Cartographic image created by the authors from the the INE's Digital Cartography Files [2] using *R 4.4.1* [3] with *ggplot2 3.4.4* [4] and *ggspatial 1.1.9* [1].

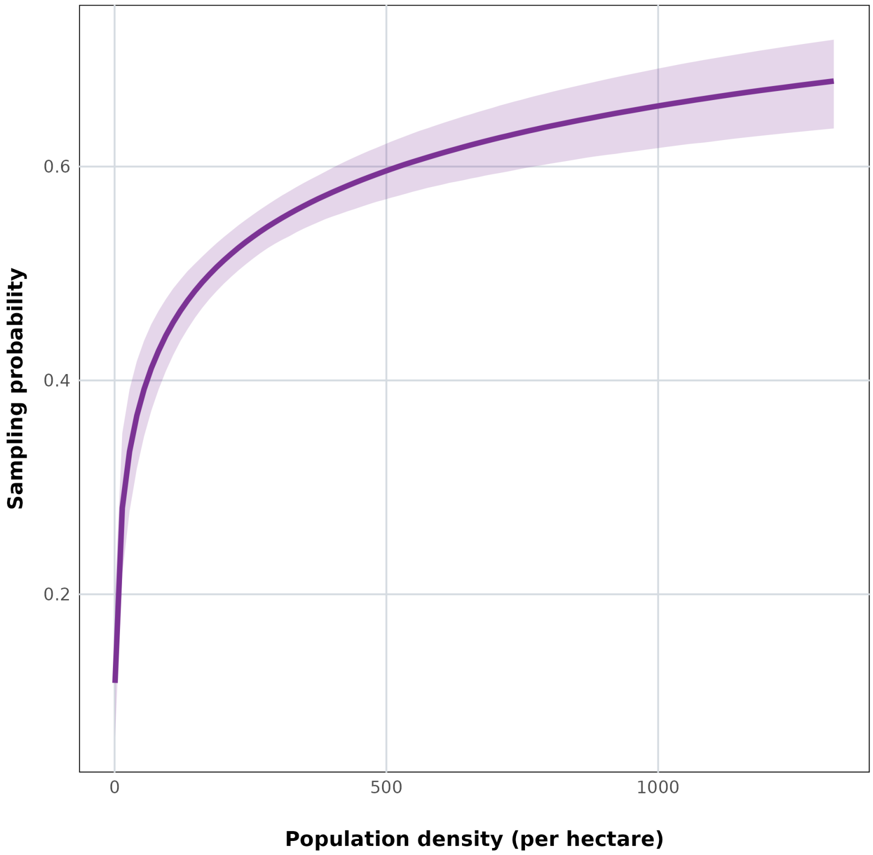

Fig. 2: Conditional effects plot of the relationship between census tract population density and predicted probability of citizen scientists reporting adult mosquitoes or mosquito bites in the Mosquito Alert General Participation Model, with all other variables held at their means.

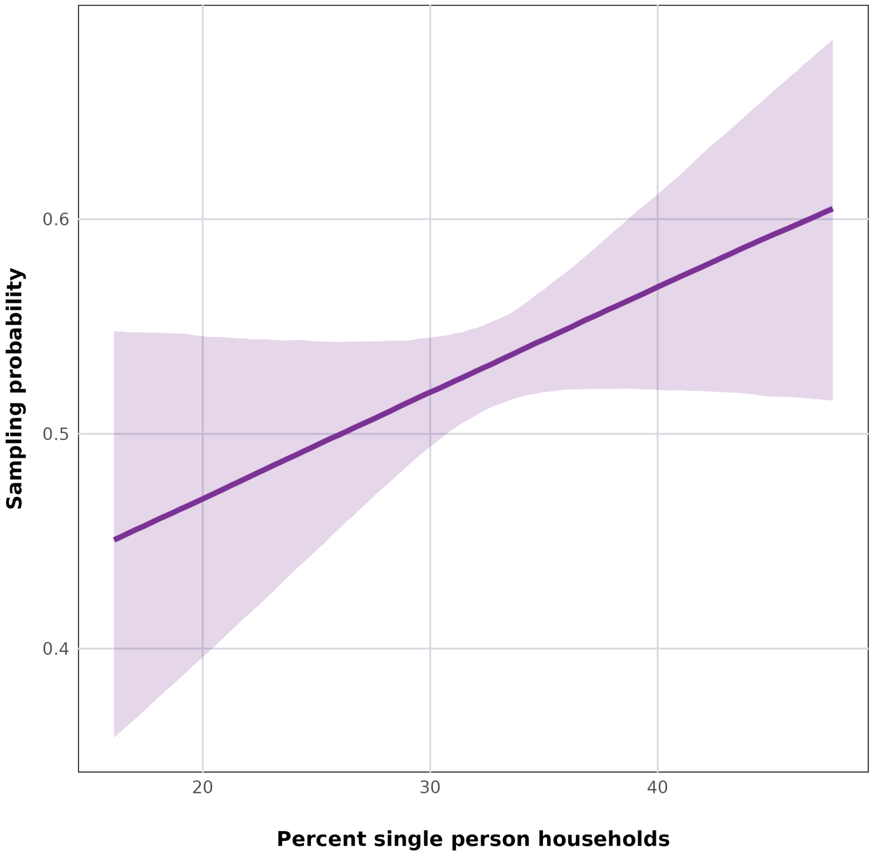

Fig. 3: Conditional effects plot of the relationship between census tract percentage of households composed of only one member and predicted probability of citizen scientists reporting adult mosquitoes or mosquito bites in the Mosquito Alert General Participation Model, with all other variables held at their means.

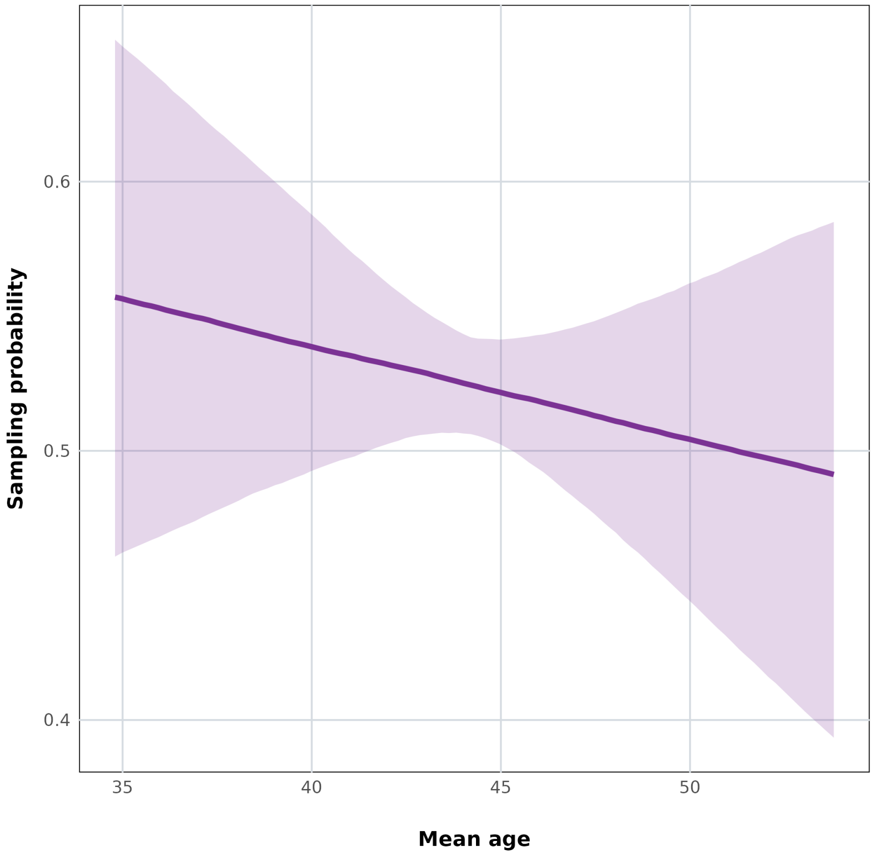

Fig. 4: Conditional effects plot of the relationship between mean age of the census tract population and predicted probability of citizen scientists reporting adult mosquitoes or mosquito bites in the Mosquito Alert General Participation Model, with all other variables held at their means.

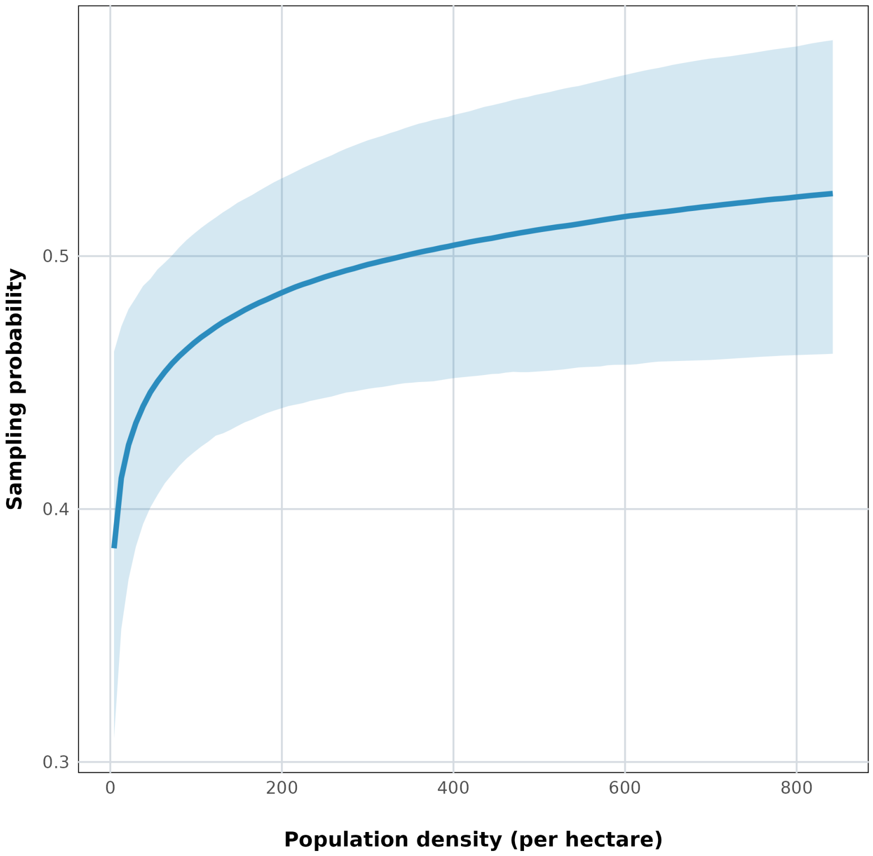

Fig. 5: Conditional effects plot of the relationship between census tract population density and predicted probability during a given year of citizen scientists reporting adult mosquitoes or mosquito bites within 200 m of a catch basin drain with known mosquito activity that year in the Active Catch Basin Drain Participation Model, with all other variables held at their means.

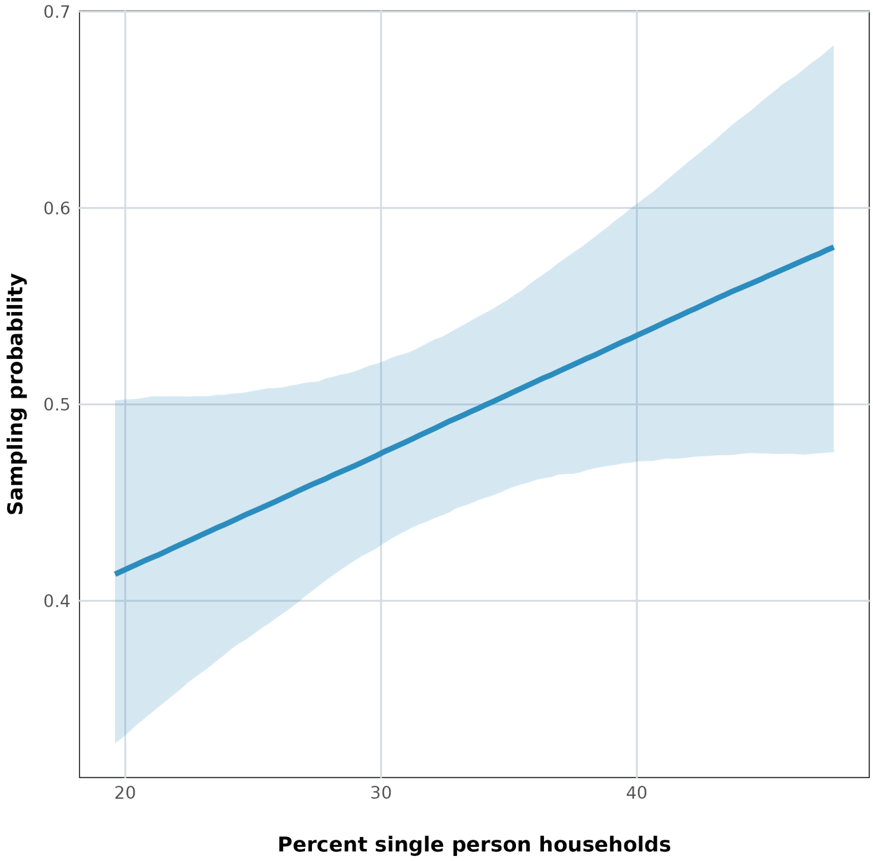

Fig. 6: Conditional effects plot of the relationship between the census section proportion of households composed of a single member and predicted probability during a given year of citizen scientists reporting adult mosquitoes or mosquito bites within 200 m of a catch basin drain with known mosquito activity that year in the Active Catch Basin Drain Participation Model, with all other variables held at their means.

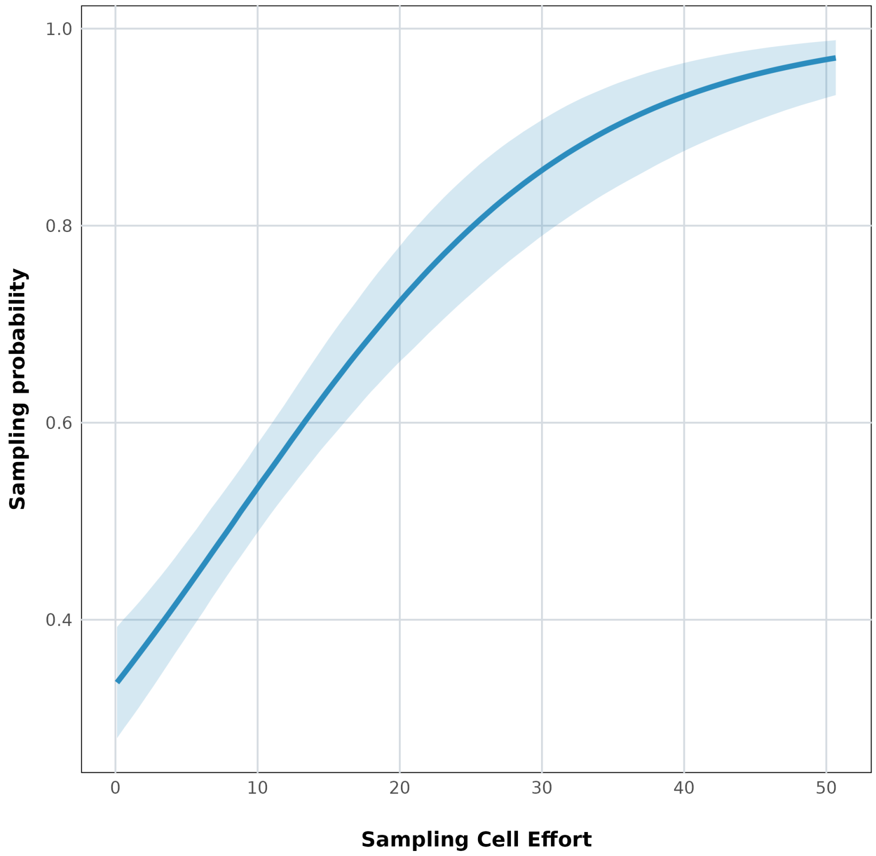

Fig. 7: Conditional effects plot of the relationship between the Mosquito Alert sampling effort estimate and predicted probability during a given year of citizen scientists reporting adult mosquitoes or mosquito bites within 200 m of a catch basin drain with known mosquito activity that year in the Active Catch Basin Drain Participation Model, with all other variables held at their means.

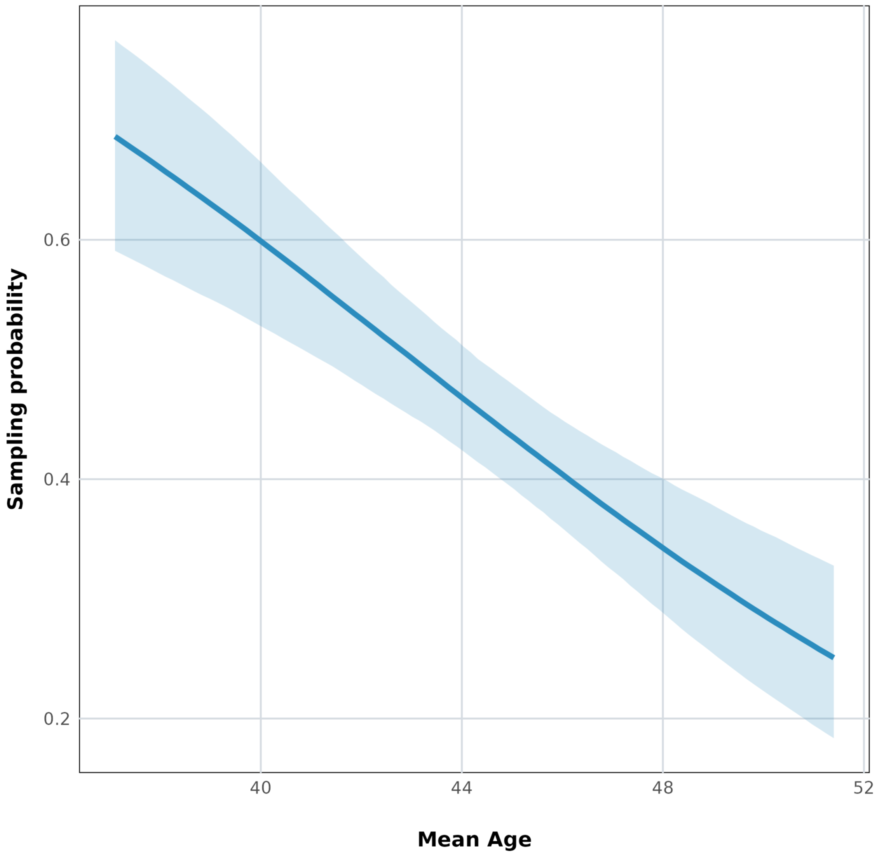

Fig. 8: Conditional effects plot of the relationship between the mean age of the census section population and predicted probability during a given year of citizen scientists reporting adult mosquitoes or mosquito bites within 200 m of a catch basin drain with known mosquito activity that year in the Active Catch Basin Drain Participation Model, with all other variables held at their means.

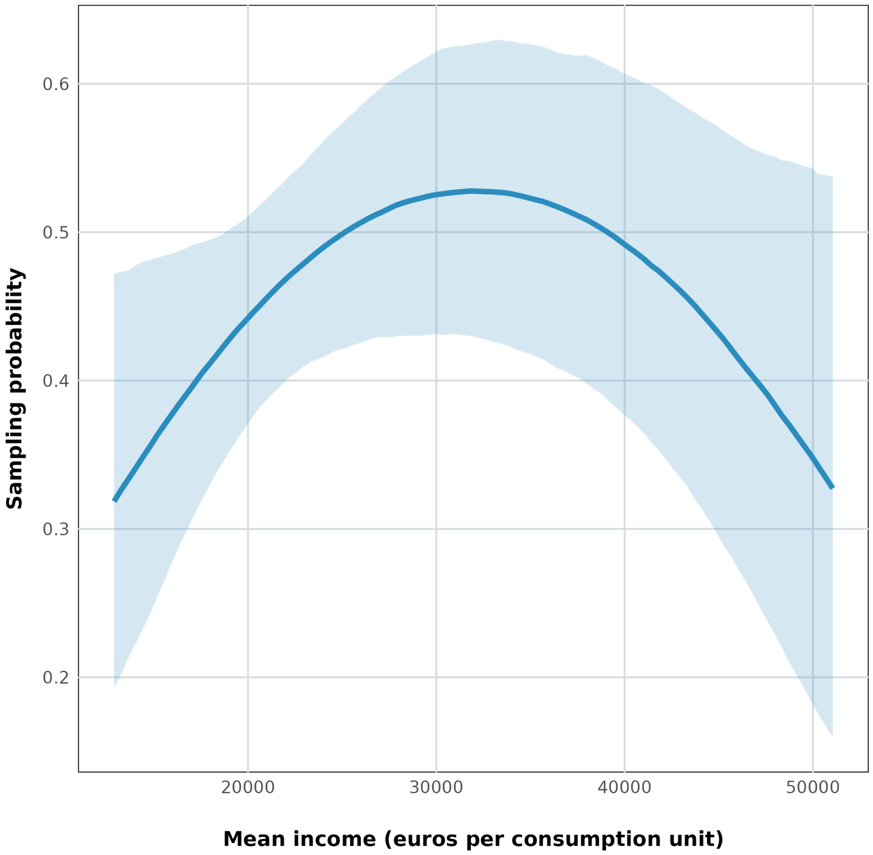

Fig. 9: Robustness check for conditional effects of the relationship between the census tract mean income per consumption unit and predicted probability during a given year of citizen scientists reporting adult mosquitoes or mosquito bites within 200 m of a catch basin drain with known mosquito activity that year in the Active Catch Basin Drain Participation Model, with all other variables held at their means. In this robustness check drains are used only if they are more than 200 m of any other drain in the sample.

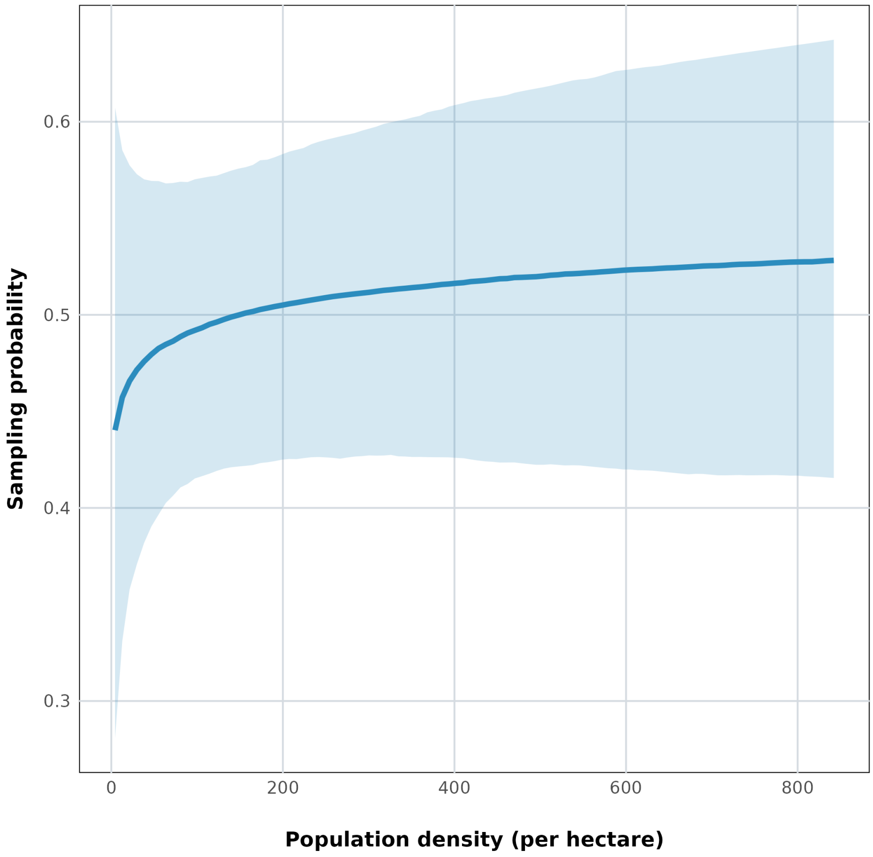

Fig. 10: Robustness check for conditional effects of the relationship between the census tract population density and predicted probability during a given year of citizen scientists reporting adult mosquitoes or mosquito bites within 200 m of a catch basin drain with known mosquito activity that year in the Active Catch Basin Drain Participation Model, with all other variables held at their means. In this robustness check drains are used only if they are more than 200 m of any other drain in the sample.

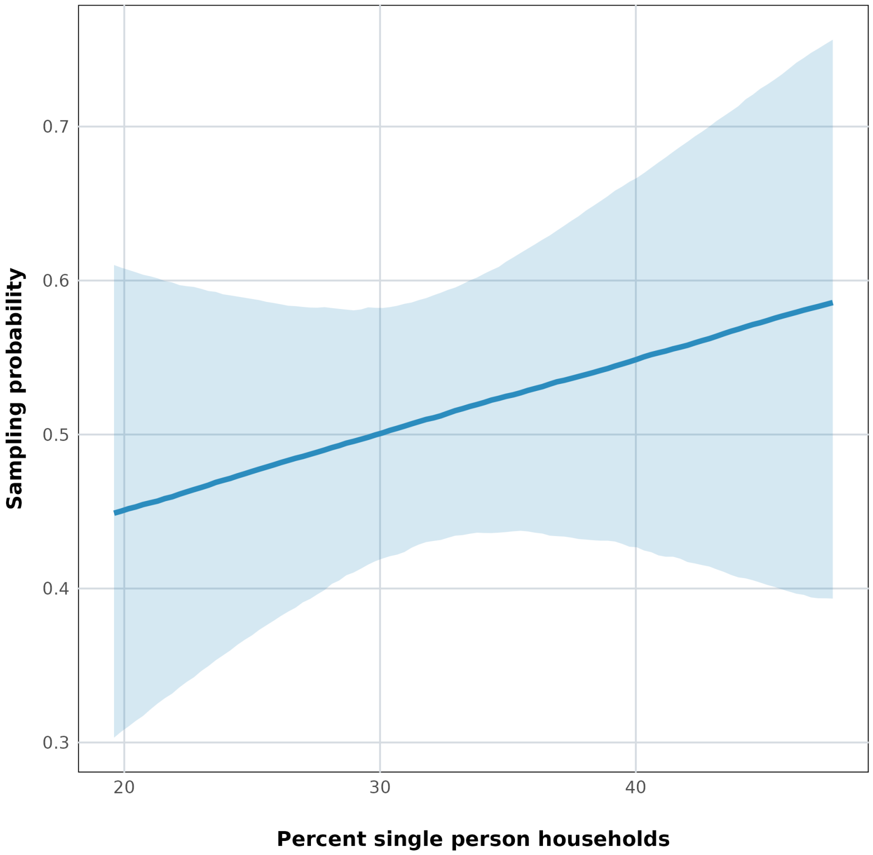

Fig. 11: Robustness check for conditional effects of the relationship between the section section proportion of households composed of a single member and predicted probability during a given year of citizen scientists reporting adult mosquitoes or mosquito bites within 200 m of a catch basin drain with known mosquito activity that year in the Active Catch Basin Drain Participation Model, with all other variables held at their means. In this robustness check drains are used only if they are more than 200 m of any other drain in the sample.

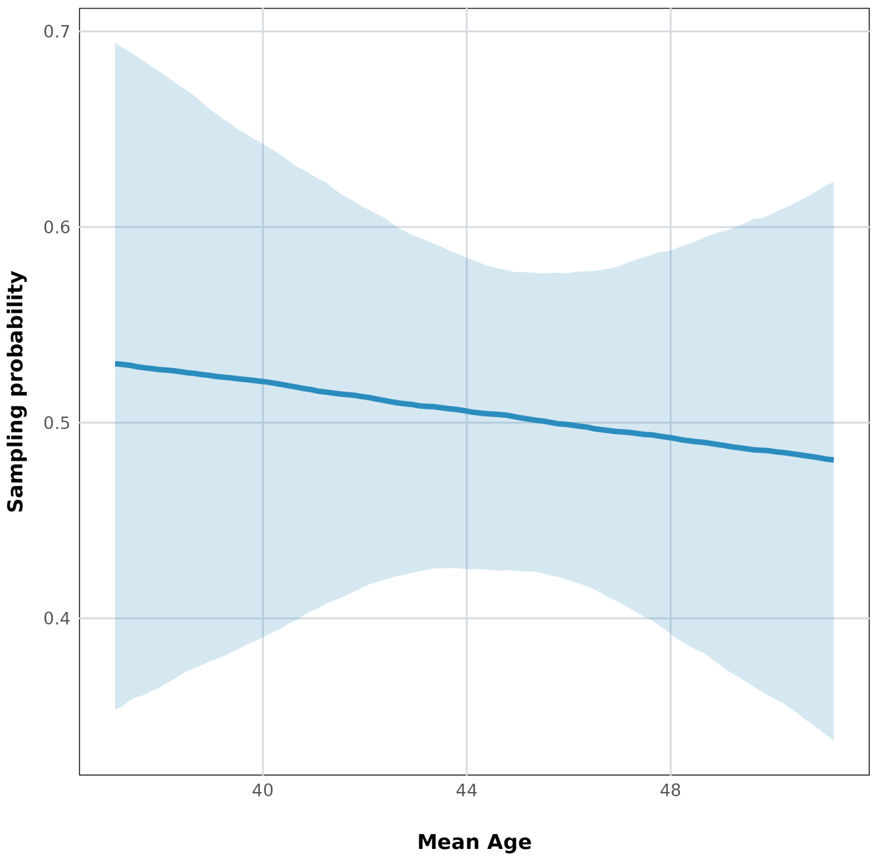

Fig. 12: Robustness check for conditional effects of the relationship between the census tract mean income per consumption unit and predicted probability during a given year of citizen scientists reporting adult mosquitoes or mosquito bites within 200 m of a catch basin drain with known mosquito activity that year in the Active Catch Basin Drain Participation Model, with all other variables held at their means. In this robustness check drains are used only if they are more than 200 m of any other drain in the sample.

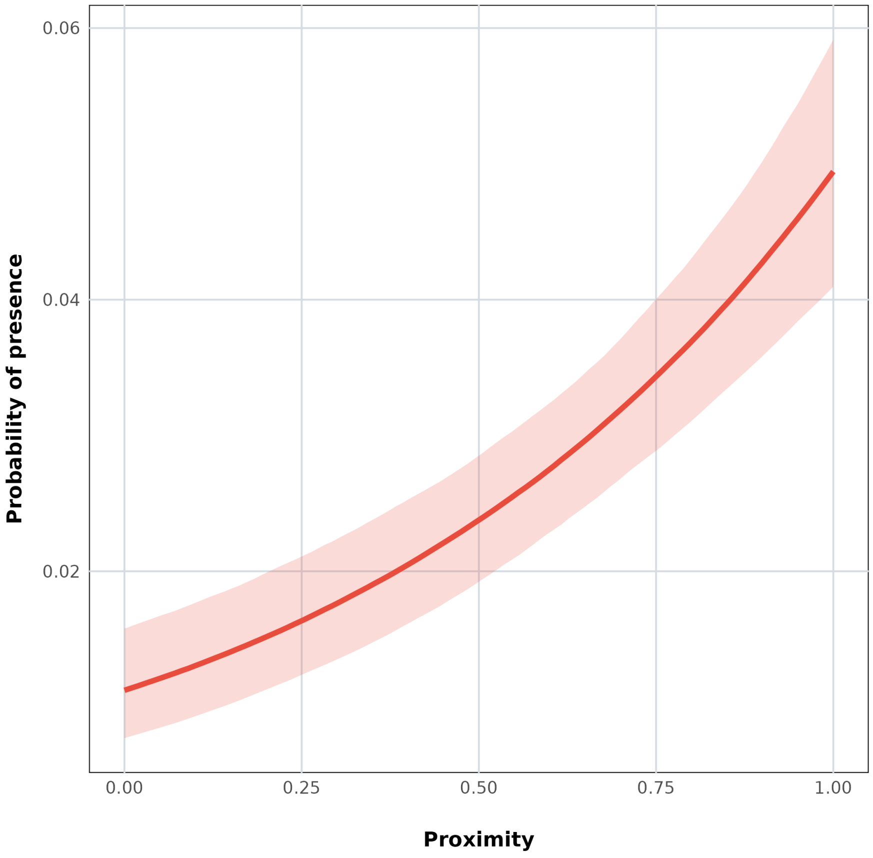

Fig. 13: Conditional effects plot of the relationship between proximity to private green space and predicted *Ae. albopictus* probability in the Mosquito Alert Vector Model, with all other variables held at their means.

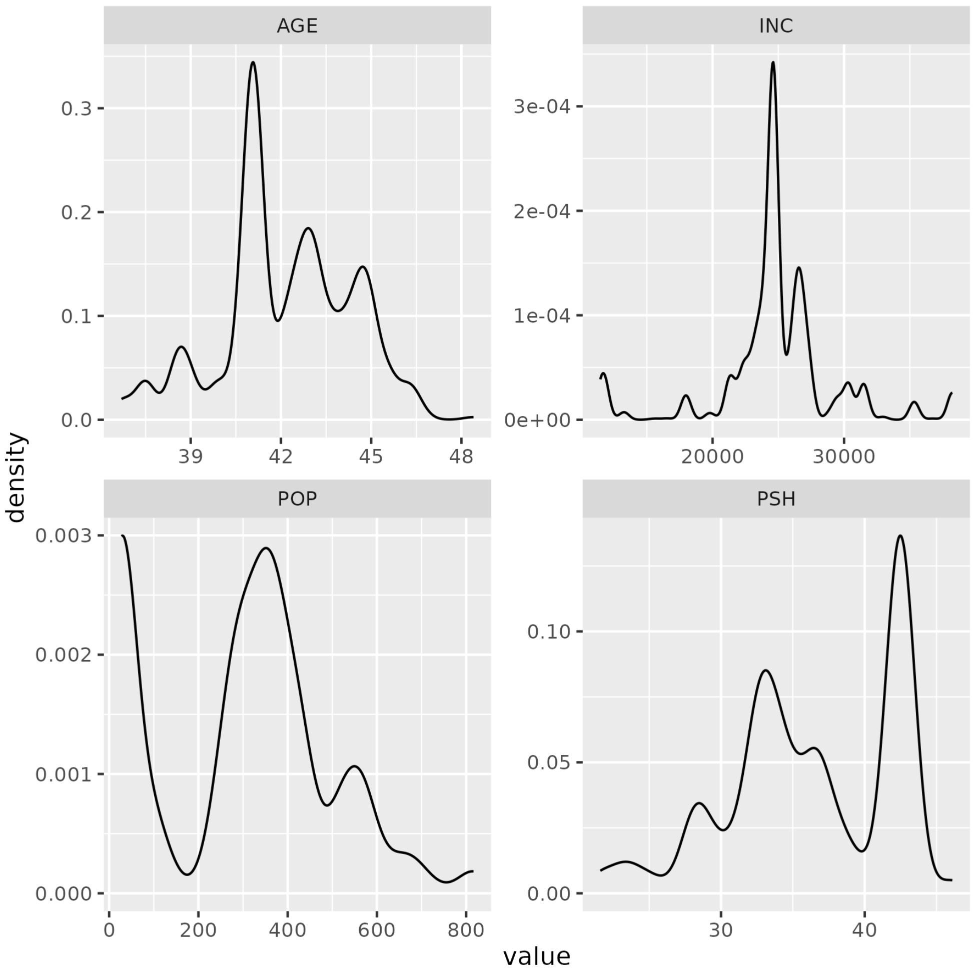

Fig. 14: Kernel density estimates of prediction points for which the model without sampling effort over-predicts by more than 10 percentage points (compared to the model with sampling effort), across the range of modeled values for: proximity to private green spaces (GPI), mean housing age (HA), mean income per consumption unit (INC), and percentage single person households (PSR).

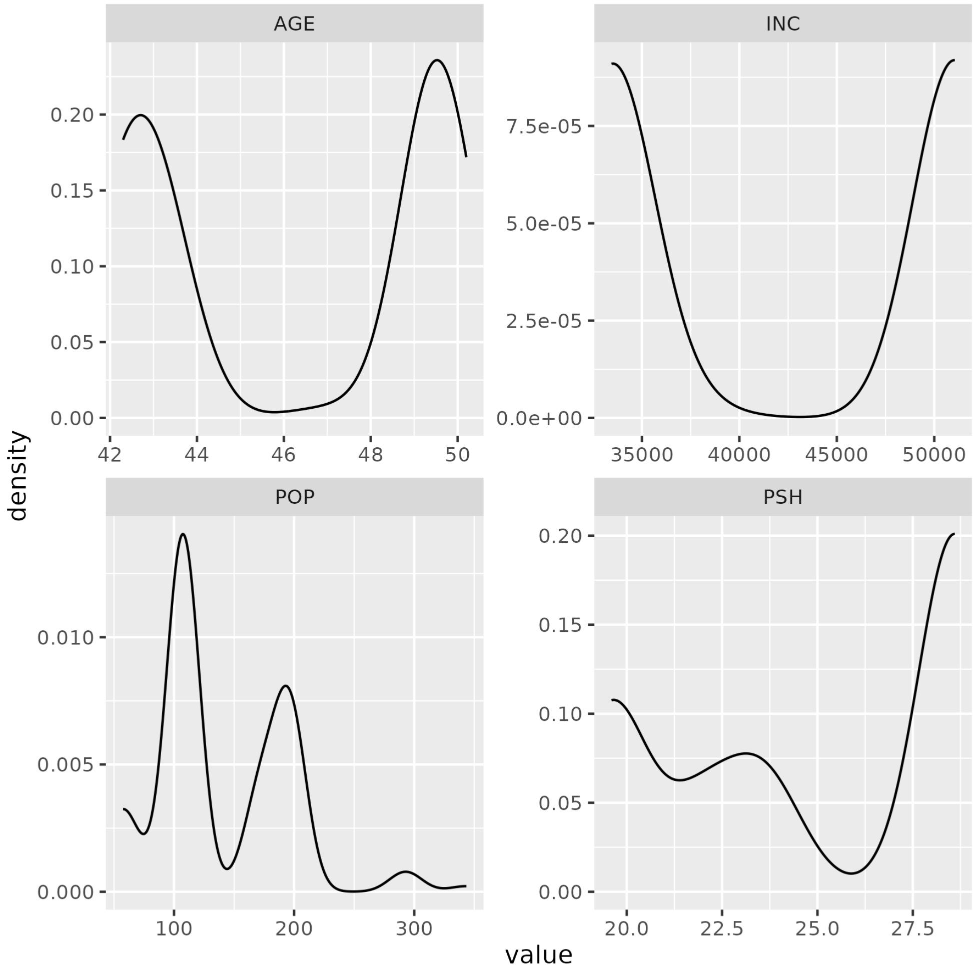

Fig. 15: Kernel density estimates of prediction points for which the model without sampling effort under-predicts by more than 10 percentage points (compared to the model with sampling effort), across the range of modeled values for: proximity to private green spaces (GPI), mean housing age (HA), mean income per consumption unit (INC), and percentage single person households (PSR).

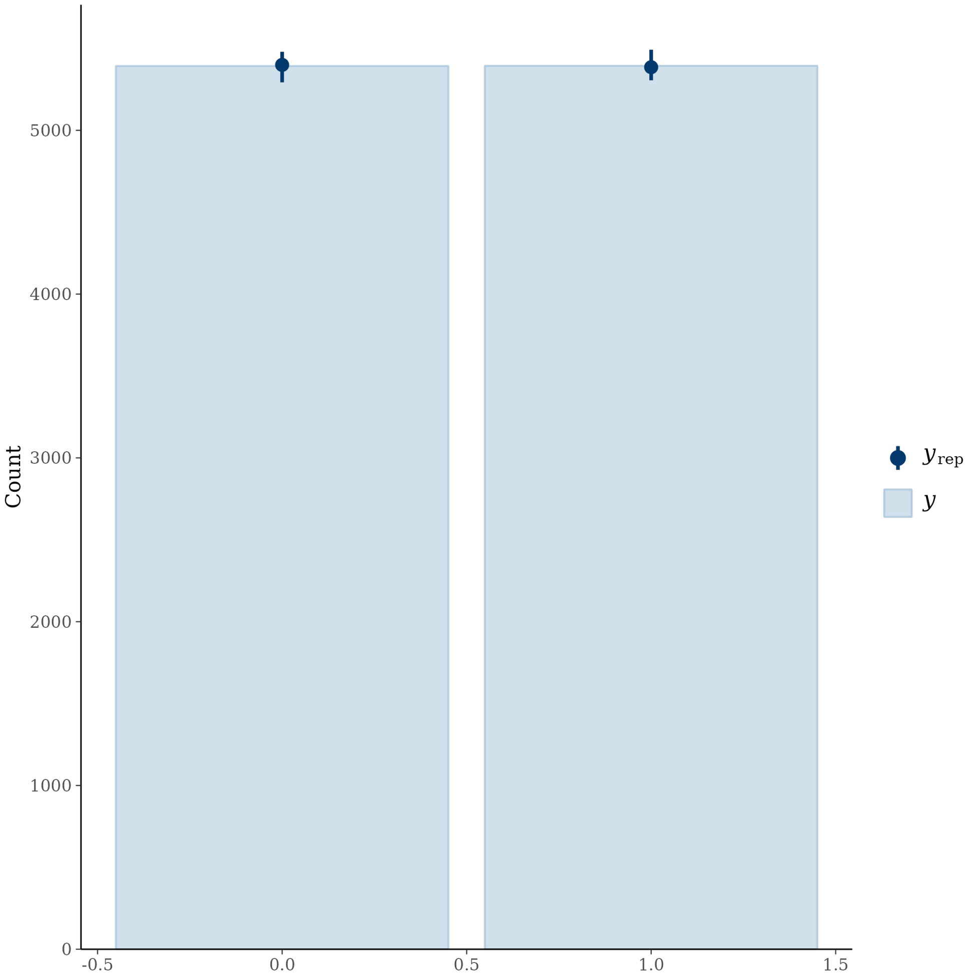

Fig. 16: Posterior predictive check of General Participation Model. Bars represent observed presences (1s) and pseudo-absences (0s) in the data. Points show the posterior predictive distribution's mean, and whiskers show the central 90% of that distribution.

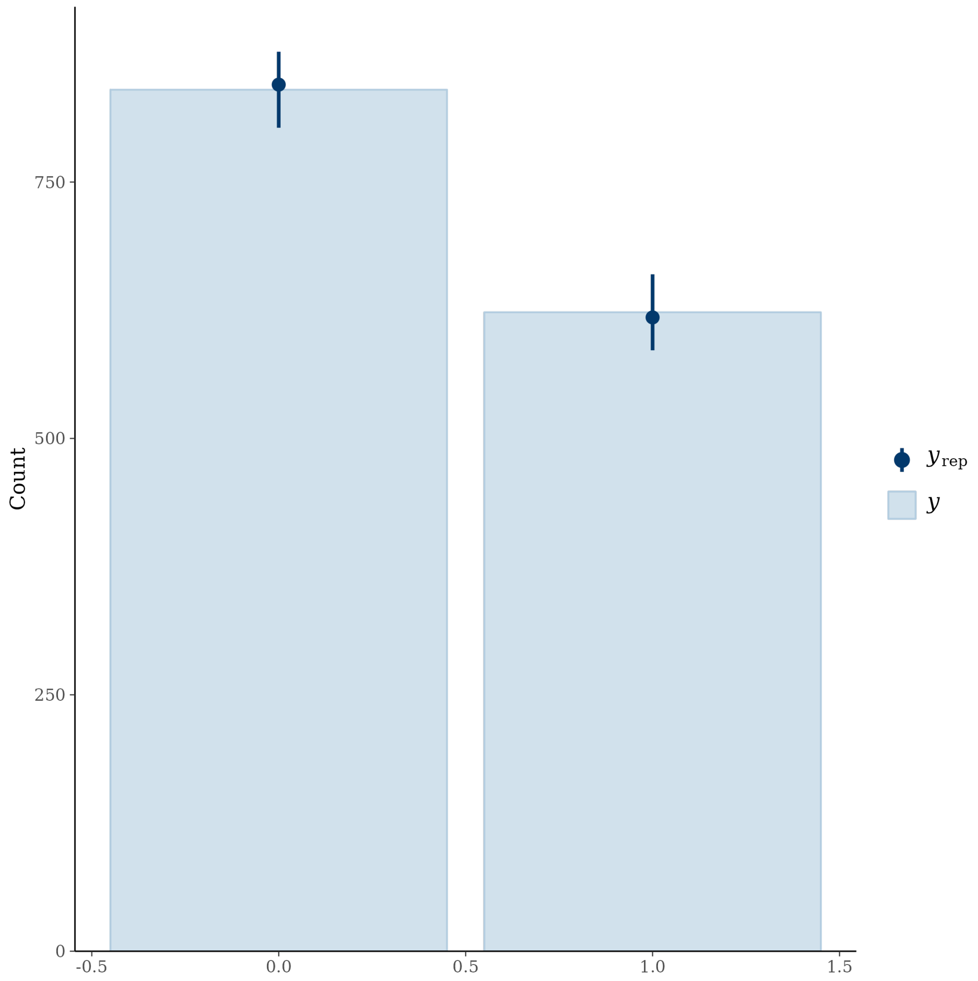

Fig. 17: Posterior predictive check of Active Catch Basin Drain Model. Bars represent observed presences (1s) and pseudo-absences (0s) in the data. Points show the posterior predictive distribution's mean, and whiskers show the central 90% of that distribution.

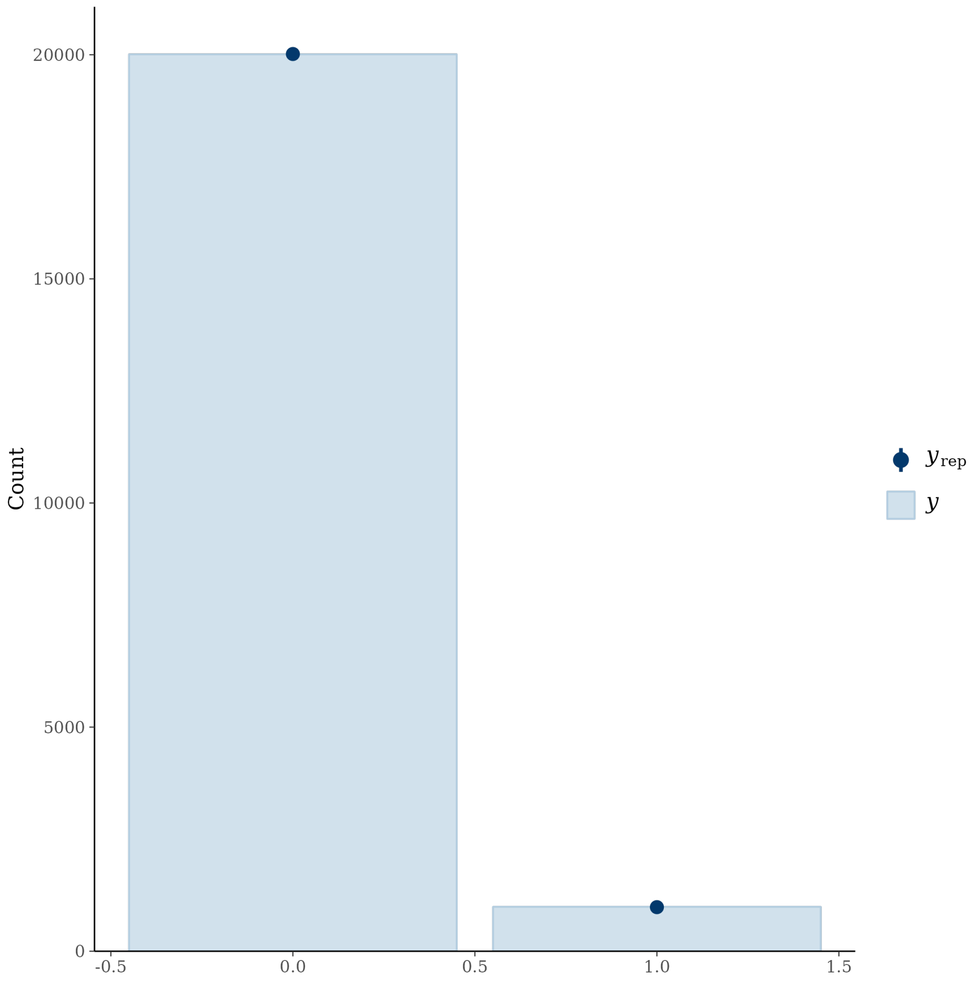

Fig. 18: Posterior predictive check of Mosquito Alert Vector Model. Bars represent observed presences (1s) and pseudo-absences (0s) in the data. Points show the posterior predictive distribution's mean, and whiskers show the central 90% of that distribution.

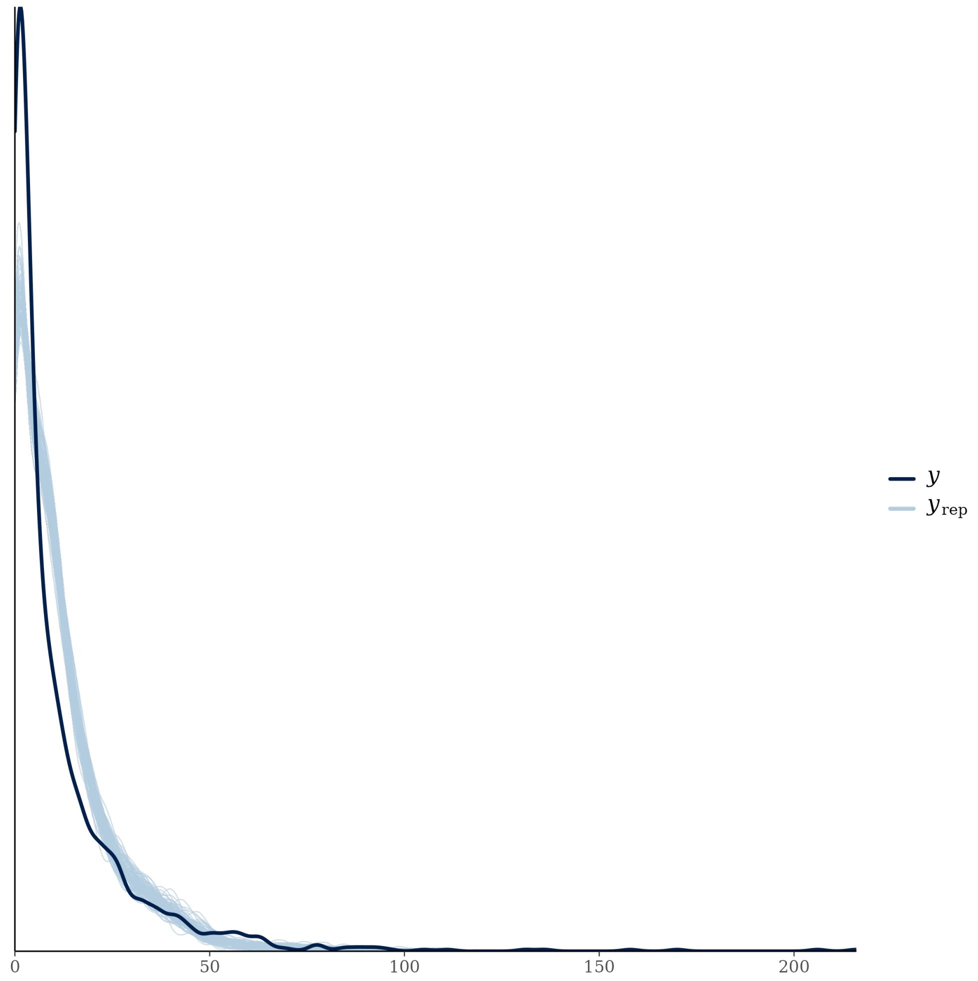

Fig. 19: Posterior predictive check of Mosquito Trap Vector Model. Dark curve shows the kernel density plot of the observed trap counts; light blue curves show kernel density plots of 100 draws from the posterior predictive distribution.

2 Supplemental Information Tables

	District	Overpredicted	Underpredicted
1	Ciutat Vella	1140	0
2	Gràcia	807	0
3	Horta-Guinardó	490	0
4	L'Eixample	220	5
5	Les Corts	68	414
6	Sant Andreu	73	0
7	Sant Martí	672	0
8	Sants-Montjuïc	421	0
9	Sarrià-Sant Gervasi	343	250

Table 1: Number of precition points at which *Ae. albopictus* probability is over-predicted and under-predcited by more then 10 percentage points when sampling effort is not accounted for (compared to the model in which it is).

Covariates	R-squared
Income	0.09
Single household	0.09
Sampling effort	NA
Mean age	0.12

Table 2: Variance of each covariate explained by the rest of covariates of the General Participation Model, estimated using a linear model

	GPM1	GPM2	GPM3	GPM4	GPM5
Int.	0.16*	−1.88*	−1.04	−1.92*	−1.97*
	[0.10; 0.22]	[−2.40; −1.38]	[−2.61; 0.46]	[−3.62; −0.14]	[−3.62; −0.27]
INC	−0.26	0.03	0.01	0.27	0.26
	[−3.62; 2.93]	[−3.13; 3.20]	[−3.23; 3.26]	[−2.97; 3.57]	[−2.89; 3.50]
INC sq.	−1.33	−1.24	−1.29	−1.25	−1.25
	[−4.36; 1.86]	[−4.43; 1.88]	[−4.53; 2.05]	[−4.38; 2.02]	[−4.39; 1.96]
POP		0.36*	0.37*	0.37*	0.37*
		[0.27; 0.45]	[0.28; 0.47]	[0.28; 0.46]	[0.28; 0.46]
AGE			−0.02	−0.01	−0.01
			[−0.05; 0.02]	[−0.05; 0.02]	[−0.05; 0.02]
PSH				0.02	0.02
				[−0.00; 0.04]	[−0.00; 0.04]
Spatial autocorr.	icar	icar	icar	none	icar
Observations	10785	10785	10785	10785	10785
Bayes R-sq.	0.36	0.35	0.35	0.35	0.35
SE Bayes R-sq.	0.01	0.01	0.01	0.01	0.01
ELPD	−5573.67	−5572.19	−5571.27	−5572.81	−5572.81
SE ELPD	51.47	51.48	51.54	51.54	51.51

* Null hypothesis value outside 90% credible interval.

Table 3: Parameter estimates for General Participation Models.

Covariates	R-squared
Income	0.16
Single household	0.26
Sampling effort	.10
Mean age	0.26

Table 4: Variance of each covariate explained by the rest of covariates of the Active Catch Basin Drain Participation Model, estimated using a linear model

	ACBDPM1	ACBDPM2	ACBDPM3	ACBDPM4	ACBDPM5	ACBDPM6
Int.	−0.31*	−0.93*	−0.97*	−2.47*	3.33*	3.55*
	[−0.41; −0.22]	[−1.11; −0.78]	[−1.29; −0.67]	[−3.19; −1.77]	[1.44; 5.24]	[1.46; 5.53]
INC	−9.63*	0.08*	0.08*	0.08*	0.08*	0.08*
	[−13.45; −5.88]	[0.07; 0.10]	[0.07; 0.10]	[0.06; 0.10]	[0.06; 0.10]	[0.07; 0.10]
INC sq.	−6.62*	−7.43*	−7.51*	−4.05	−5.65*	−5.81*
	[−10.40; −3.09]	[−11.27; −3.56]	[−11.50; −3.50]	[−8.34; 0.30]	[−9.68; −1.65]	[−10.10; −1.57]
SE		−2.77	−2.55	−2.73	−5.07*	−5.37*
		[−6.41; 1.11]	[−6.46; 1.21]	[−6.69; 1.17]	[−8.98; −1.24]	[−9.34; −1.44]
POP			0.01	0.02	0.11*	0.11*
			[−0.06; 0.08]	[−0.05; 0.09]	[0.03; 0.17]	[0.03; 0.18]
PSH				0.05*	0.03*	0.02*
				[0.03; 0.07]	[0.00; 0.05]	[0.00; 0.05]
AGE					−0.13*	−0.13*
					[−0.17; −0.09]	[−0.17; −0.09]
Random Intercepts	drain	drain	drain	drain	none	drain
Observations	1463	1463	1463	1463	1463	1463
Bayes R-sq.	0.04	0.12	0.12	0.12	0.11	0.14
SE Bayes R-sq.	0.03	0.04	0.04	0.03	0.01	0.03
ELPD	−987.75	−941.06	−941.69	−935.21	−920.62	−921.81
SE ELPD	7.89	12.16	12.15	12.90	13.99	14.01

* Null hypothesis value outside 90% credible interval.

Table 5: Parameter estimates for Active Catch Basin Drain Participation Models.

	MAVM1	MAVM2	MAVM3	MAVM4
Int.	−2.34*	−3.43*	−3.40*	−3.47*
	[−2.46; −2.21]	[−3.72; −3.14]	[−3.59; −3.20]	[−3.77; −3.19]
INC	69.90*		23.03*	62.93*
	[33.77; 103.21]		[13.64; 32.20]	[31.38; 94.72]
INC sq.	−23.59*		−55.01*	−24.20*
	[−44.08; −2.05]		[−65.45; −45.10]	[−45.21; −3.16]
GPI		1.56*	2.01*	1.52*
		[1.21; 1.90]	[1.77; 2.25]	[1.19; 1.87]
Spatial Autocorr.	icar	icar	none	icar
Observations	21000	21000	21000	21000
Bayes R-sq.	0.21	0.21	0.03	0.21
SE Bayes R-sq.	0.01	0.01	0.00	0.01
ELPD	−2737.90	−2714.29	−3245.23	−2711.81
SE ELPD	69.56	68.62	74.22	68.59

* Null hypothesis value outside 90% credible interval.

Table 6: Parameter estimates for Mosquito Alert Vector Models.

	MTVM1	MTVM2	MTVM3	MTVM4
Int.	1.79*	1.94*	2.51*	1.96*
	[1.48; 2.12]	[1.63; 2.23]	[2.50; 2.53]	[1.67; 2.26]
TMP	18.53*	17.49*	15.66*	17.50*
	[17.89; 19.21]	[16.81; 18.15]	[15.03; 16.35]	[16.82; 18.16]
TMP sq.	−5.38*	−3.71*	−3.85*	−3.74*
	[−6.01; −4.79]	[−4.34; −3.09]	[−4.45; −3.27]	[−4.37; −3.12]
INC	1.48*		2.88*	1.65*
	[0.06; 2.92]		[2.41; 3.35]	[0.15; 3.12]
INC sq.	−0.63		−0.32	−0.60
	[−2.19; 1.00]		[−0.80; 0.18]	[−2.14; 0.88]
ZI Int.		−2.35*	−1.69*	−2.35*
		[−2.54; −2.17]	[−1.82; −1.57]	[−2.54; −2.16]
ZI TMP		−9.62*	−11.87*	−9.66*
		[−14.93; −4.60]	[−16.08; −7.67]	[−14.82; −4.43]
ZI TMP sq.		22.99*	17.98*	23.10*
		[17.57; 28.46]	[13.66; 22.11]	[17.66; 28.50]
Random Intercepts	trap	trap	none	trap
Distribution	Poisson	ZI Poisson	ZI Poisson	ZI Poisson
Observations	1368	1368	1368	1368
Bayes R-sq.	0.37	0.37	0.05	0.37
SE Bayes R-sq.	0.01	0.01	0.00	0.01
ELPD	−8569.92	−7707.36	−11605.41	−7692.55
SE ELPD	411.42	357.50	702.71	354.52

* Null hypothesis value outside 90% credible interval.

Table 7: Parameter estimates for Mosquito Trap Vector Models.

References

[1] Dunnington D (2023) ggspatial: Spatial Data Framework for ggplot2. URL https://CRAN.R-project.org/package=ggspatial, r package version 1.1.9

[2] INE (2022) Cartografía digitalizada de secciones censales. URL https://www.ine.es/dyngs/INEbase/es/operacion.htm?c=Estadistica_C&cid=1254736177088&menu=resultados&idp=1254735976608

[3] R Core Team (2023) R: A Language and Environment for Statistical Computing. R Foundation for Statistical Computing, Vienna, Austria, URL https://www.R-project.org/

[4] Wickham H (2016) ggplot2: Elegant Graphics for Data Analysis. Springer-Verlag New York, URL https://ggplot2.tidyverse.org
